# Systemic antibody responses against gut microbiota flagellins implicate shared and divergent immune reactivity in Crohn’s disease and chronic fatigue syndrome

**DOI:** 10.1186/s40168-024-01858-1

**Published:** 2024-07-29

**Authors:** Arno R. Bourgonje, Nicolai V. Hörstke, Michaela Fehringer, Gabriel Innocenti, Thomas Vogl

**Affiliations:** 1 The Henry D. Janowitz Division of Gastroenterology, Department of Medicine, Icahn School of Medicine at Mount Sinai, New York, NY USA; 2 Department of Gastroenterology and Hepatology, University of Groningen, University Medical Center Groningen, Groningen, the Netherlands; 3 Center for Cancer Research, Medical University of Vienna, Vienna, Austria

## Abstract

**Background:**

Elevated systemic antibody responses against gut microbiota flagellins are observed in both Crohn’s disease (CD) and myalgic encephalomyelitis/chronic fatigue syndrome (ME/CFS), suggesting potential serological biomarkers for diagnosis. However, flagellin-specific antibody repertoires and functional roles in the diseases remain incompletely understood. Bacterial flagellins can be categorized into three types depending on their interaction with toll-like receptor 5 (TLR5): (1) “stimulator” and (2) “silent” flagellins, which bind TLR5 through a conserved N-terminal motif, with only stimulators activating TLR5 (involving a C-terminal domain); (3) “evader” flagellins of pathogens, which entirely circumvent TLR5 activation via mutations in the N-terminal TLR5 binding motif.

**Results:**

Here, we show that both CD and ME/CFS patients exhibit elevated antibody responses against distinct regions of flagellins compared to healthy individuals. N-terminal binding to *Lachnospiraceae* flagellins was comparable in both diseases, while C-terminal binding was more prevalent in CD. N-terminal antibody-bound flagellin sequences were similar across CD and ME/CFS, resembling “stimulator” and “silent” flagellins more than evaders. However, C-terminal antibody-bound flagellins showed a higher resemblance to the stimulator than to silent flagellins in CD, which was not observed in ME/CFS.

**Conclusions:**

These findings suggest that antibody binding to the N-terminal domain of stimulator and silent flagellins may impact TLR5 activation in both CD and ME/CFS patients. Blocking this interaction could lead commensal bacteria to be recognized as pathogenic evaders, potentially contributing to dysregulation in both diseases. Furthermore, elevated antibody binding to the C-terminal domain of stimulator flagellins in CD may explain pathophysiological differences between the diseases. Overall, these results highlight the diagnostic potential of these antibody responses and lay a foundation for deeper mechanistic studies of flagellin/TLR5 interactions and their impact on innate/adaptive immunity balance.

**Graphical Abstract:**

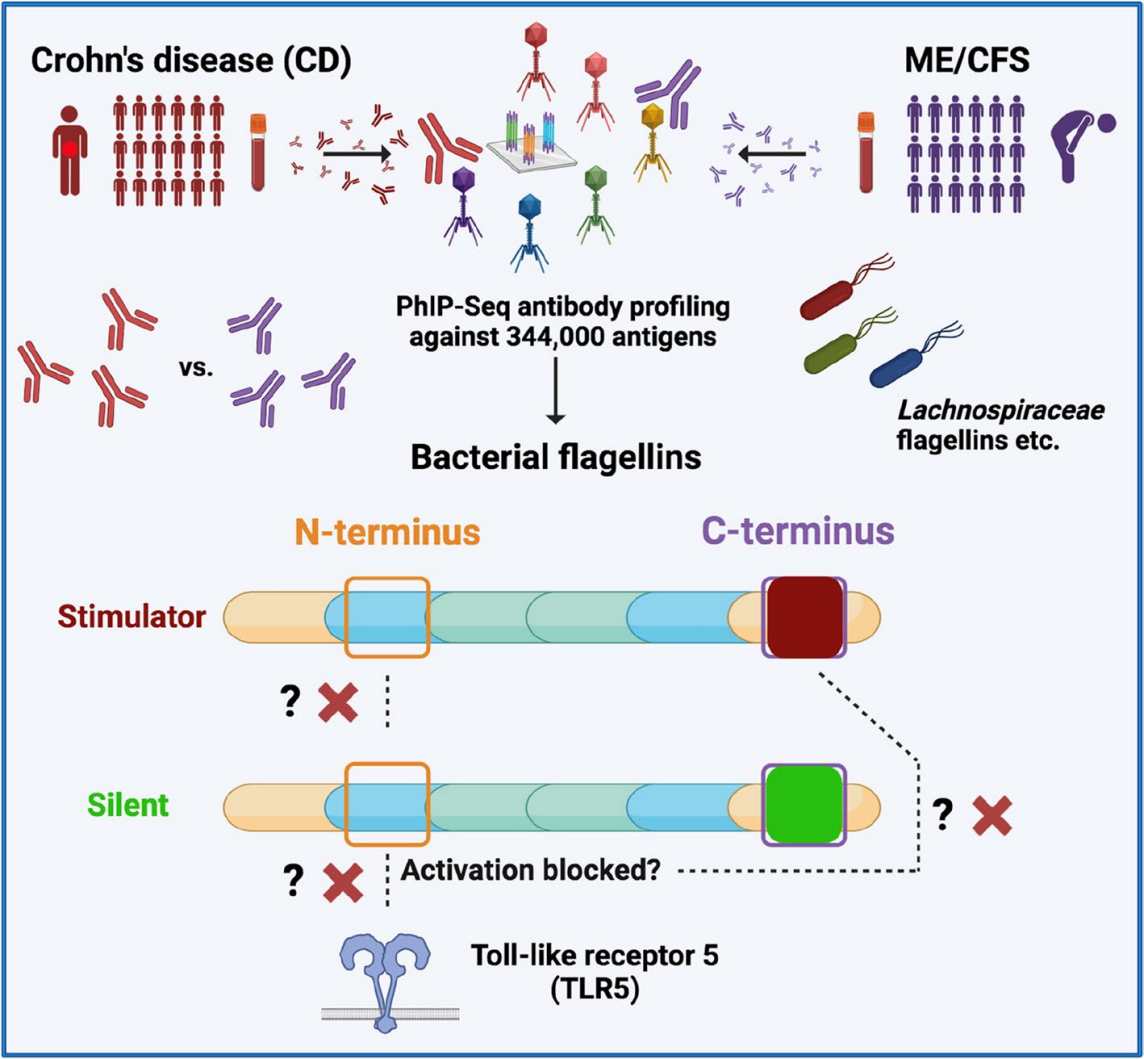

Video Abstract

**Supplementary Information:**

The online version contains supplementary material available at 10.1186/s40168-024-01858-1.

## Introduction

**Fig. 1:**
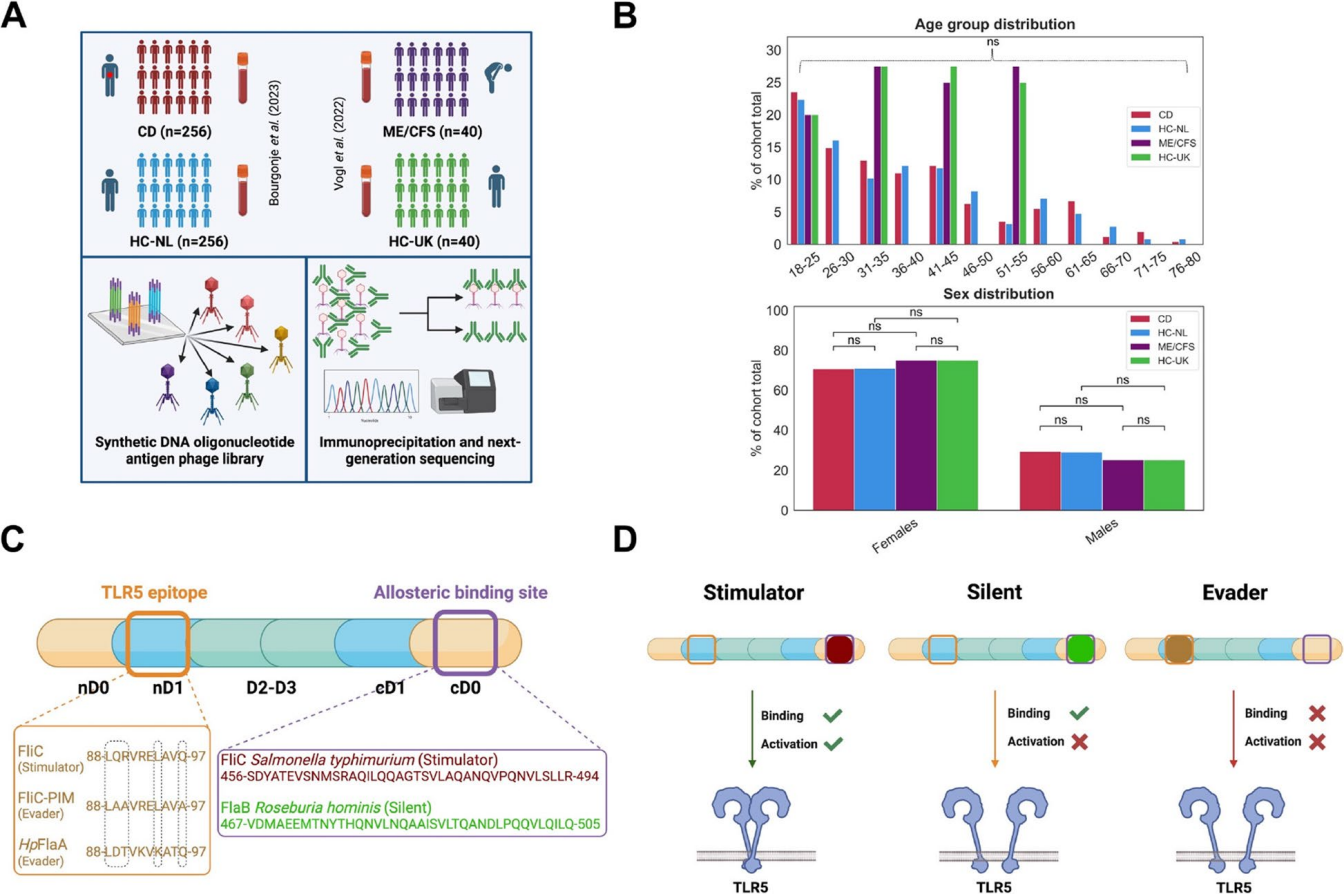
In-depth characterization of antibody repertoires in CD [10] and ME/CFS [11] yields detailed information on antibody specificities against bacterial flagellins, which can be classified into three types depending on their interaction with TLR5 [12]. **A** PhIP-Seq was used to characterize antibody epitope repertoires against a breadth of peptide antigens (as previously described [13], see main text) in patients with CD and matched controls from the Netherlands (denoted as HC-NL) [10], as well as in patients with ME/CFS and matched controls from the U.K. (denoted as HC-UK) [11]. **B** Age- and sex distributions are not significantly different across all cohorts (age: Wilcoxon tests, *P* = 0.614 and *P* = 0.921 for CD vs. HC-NL and ME/CFS vs. HC-UK; sex: chi-square test, *P* = 0.899). **C** Flagellin domain structure shows the TLR5 motif in the N-terminal D1 (nD1) domain and the allosteric binding site located in the C-terminal D0 (cD0) domain. Multiple sequence alignment of the nD1 TLR5 motif for three sample flagellins is shown (FliC, a *S. typhimurium* flagellin, classified as ‘stimulator’; FliC-PIM, FliC with mutated key residues in the TLR5 motif, reducing TLR5 binding compared to FliC; *Hp*FlaA, flagellin from *H. pylori* with different amino acids in the TLR5 motif site, also classified as ‘evader’) alongside two sample cD0 amino acid sequences derived from Clasen et al. [12] (FliC and FlaB, a representative ‘silent’ flagellin from *Roseburia hominis* [12]). Dotted lines surround the key residues required for TLR5 motif binding. **D** Flagellin categorization described by Clasen et al. [12], depending on TLR5 binding and allosteric binding

Homeostatic mechanisms contributing to establishing immunological tolerance towards non-harmful microbial antigens usually prevent systemic immune activation against the human gut microbiota [1]. Disturbances in the intricate equilibrium between the gut microbiota and host immune system have been linked to various diseases, including inflammatory bowel diseases (IBDs) such as Crohn’s disease (CD) and ulcerative colitis [2, 3], as well as myalgic encephalomyelitis/chronic fatigue syndrome (ME/CFS) [4]. In both CD and ME/CFS, dysbiosis of the gut microbiota, disrupted gut barrier integrity, and increased bacterial translocation have been implied as pathological manifestations [5, 6]. Notably, in CD [7–10] and recently also ME/CFS [11] elevated systemic antibody responses against gut microbiota flagellins have been reported (Fig. 1a, b [10, 11]). Although these antibodies have been suggested as serological biomarkers for these diseases, their flagellin-specific antibody repertoires and functional roles remain incompletely understood.

Bacterial flagellins constitute widely recognized microbial antigens and are the building blocks of flagella facilitating movement toward favorable microenvironments, such as the mucus layer that lines the intestinal epithelium [14]. Flagellins possess a protein structure comprising conserved amino (N)- and carboxy (C)-terminal domains polymerizing internally within the flagellum (Fig. 1c). These domains flank a hypervariable region that faces the exterior [15]. Flagellins exhibit remarkable antigenic potency, capable of activating both immune and non-immune cells, thereby eliciting innate and adaptive immune responses. Most notably, flagellins are the only protein group known to activate toll-like receptors (key players in innate immune activation) in a sequence-specific manner [16]: Toll-like receptor 5 (TLR5), a receptor abundantly expressed in the intestinal epithelium and lamina propria, binds to a conserved N-terminal motif of flagellins (the “TLR5 motif” located in the conserved N-terminal D1 domain, nD1-Fig. 1c) and thereby plays a key role in regulating immune responses induced by flagellin [17]. Furthermore, TLR5 is found in T cells but lacking in most B cells [17–19], except for B cells in Peyer’s patches [20–22], highlighting its complex involvement in the regulation of adaptive immune responses, resulting in the generation of flagellin-specific T cells and antibody production. Flagellated bacteria are however not exclusively composed of potentially pathogenic species such as *Salmonella* [23]–they are also commonly found on commensal gut microbiota with most flagellated commensals belonging to the Firmicutes phylum and the *Lachnospiraceae* family [9].

Recently, it has been shown that such bacterial flagellins can be categorized into three classes depending on their toll-like receptor (TLR5) signaling modulation [12]: (1) “stimulator”, (2) “silent”, and (3) “evader” flagellins. The *Salmonella typhimurium*-derived FliC flagellin serves as a notable example of stimulator flagellins, demonstrating high ligand potency and leading to TLR5 dimerization and activation [24]. Conversely, mutations in the crucial residues of the nD1 domain (FliC PIM) result in an approximately nine-fold reduction in binding, thereby abrogating TLR5 activation [12]. ‘Evader’ flagellins lack the ability to activate TLR5 due to variations in the amino acids within their TLR5 motif, preventing recognition by TLR5 and subsequent receptor activation [25]. The FlaA flagellin from pathogenic bacteria such as *Helicobacter pylori* (*Hp*FlaA) serves as a notable example. The ‘silent’ class of flagellins is characterized by the preservation of the N-terminal TLR5 motifs while featuring alterations in the conserved C-terminal D0 domain (cD0, harboring an allosteric binding site for TLR5) [12]. In the case of “stimulator” flagellins, this cD0 domain contributes to TLR5 activation independently of direct binding of the N-terminal TLR5 motif to TLR5. Silent flagellins are weak TLR5 agonists that retain binding to the receptor via the N-terminal nD1 domain. Silent flagellins lack a TLR5-binding site in the cD0 domain that, in the case of the stimulator FliC, acts as an allosteric activator of TLR5 [26].

Notably, elevated antibody responses directed against flagellated bacteria, particularly those belonging to the *Lachnospiraceae* family [9, 10] have been observed in CD and ME/CFS [7, 8, 11]. In the case of CD, certain anti-flagellin antibodies, such as anti-CBir1, anti-Fla-X, and anti-A4-Fla2, have shown potential as biomarkers for the disease [27, 28]. Likewise, anti-flagellin antibodies have been suggested as diagnostic markers in ME/CFS [11]. However, while patients with CD have a higher risk of developing ME/CFS [29], most patients with CD do not experience the profound fatigue and post-exertional malaise commonly observed in ME/CFS patients (although patients with CD do often report fatigue [30]). Similarly, severe gastrointestinal (GI) inflammation characteristic of CD is not frequently observed in ME/CFS (albeit patients do report GI complaints) [4]. Given the systemic antibody responses against flagellated bacteria observed in both CD and ME/CFS, the question arises as to which specific types of flagellins (stimulator, evader, or silent flagellins) and to what regions of the flagellin protein (such as the N-terminal nD1-domain containing TLR5 motif or the C-terminal D0 domain-located allosteric binding site essential for receptor activation) these antibodies are directed. It appears plausible that antibody binding to the N-terminal portion of flagellin peptides may impede their binding to TLR5, while antibody binding to the C-terminal part of flagellin peptides may interfere with the allosteric binding site required for reinforcing TLR5 activation (Fig. 1c).

In this study, we compared anti-flagellin antibody responses in patients with CD and ME/CFS that were generated using a state-of-the-art high-throughput and high-resolution antibody repertoire profiling technique (phage-display immunoprecipitation sequencing, PhIP-Seq [10, 11]). We show that patients with CD and ME/CFS display shared and divergent antibody responses against N-terminal vs. C-terminal regions of flagellins including distinct patterns of binding against stimulator-, silent-, and evader-type flagellins.

## Materials and methods

### Study cohorts and data

#### 1000IBD cohort

Serum samples were previously obtained from 256 individuals with a confirmed diagnosis of CD and put forward for PhIP-Seq profiling [10]. These participants were included in the University Medical Center Groningen (UMCG) as part of their involvement in the 1000IBD project, which represents a substantial multi-omics IBD cohort based in Groningen, the Netherlands [31]. The recruitment of these individuals spanned from 2007 to 2019, during which comprehensive phenotypic data and multi-omics information were obtained. All clinical assessments were performed at the time of sample collection. All participants were granted written informed consent prior to their samples being collected. Ethical clearance for the study was obtained from the Institutional Review Board (IRB) of the UMCG (IRB no. 2008/338), adhering to the principles outlined in the Declaration of Helsinki (2013).

#### Lifelines-DEEP cohort

Additionally, plasma samples derived from a population-based cohort known as Lifelines-DEEP, located in the northern region of the Netherlands, were used for PhIP-Seq profiling. This cohort constitutes a section of Lifelines, a prospective multi-generational population-based study that investigates health and health-related behaviors over time among more than 160,000 individuals residing in the northern region of the country [32]. This dataset encompasses demographic, biomedical, socio-economic, behavioral, physical, and psychological factors, all contributing to the understanding of health and disease patterns in the general population. From this Lifelines-DEEP dataset, data from 256 individuals (age- and sex-matched to CD patients) were included in this specific study [10, 33] after excluding those with known irritable bowel syndrome (IBS) or IBD. Ethical approval for the Lifelines-DEEP cohort study was also granted by the IRB of the UMCG (IRB no. M12.113965) and registered at the Lifelines Research Site in Groningen. Further comprehensive details regarding this cohort can be found elsewhere [33].

#### ME/CFS cohort and healthy controls

Serum samples were acquired from two distinct groups comprising 40 severe cases of ME/CFS and 40 age- and sex-matched healthy controls, sourced from the United Kingdom Myalgic Encephalomyelitis Biobank (UKMEB) [34]. Healthy controls were matched in a 1:1 ratio to ME/CFS patients, considering the potential impact of age and sex on antibody epitope repertoires [13]. Data on age was only available in groups spanning five years. Notably, these healthy controls were also recruited through the UKMEB, effectively mitigating any potential biases associated with geographical or sample handling differences (specific identities of the matched pairs were not disclosed). All research involving these serum samples received ethical approval from the IRB of the Weizmann Institute of Science (Rehovot, Israel) (IRB no. #1410–2) and the donors had provided explicit consent for the use of their donated samples.

### PhIP-Seq procedure

#### Antigen libraries

Both in the CD and ME/CFS studies, two antigen libraries were utilized [13, 33, 35], together comprising a comprehensive collection of 344,000 antigens. The first library, covering 244,000 variants, encompasses a diverse range of primarily microbial antigens derived from 28,668 different proteins. Among these, the majority (27,837 proteins) originate from microbiota antigens. This library includes antigens from various sources, such as pathogenic bacteria (~ 10%), probiotic bacteria (~ 6%) [36], known antibody-coated bacteria (~ 9%) [37], and genes and strains of commensal bacteria based on antigen selection from metagenomic sequencing of nearly 1000 healthy individuals (~ 60%) [38]. Additionally, bacterial virulence factor antigens (~ 10%) were retrieved from the virulence factor database (VFDB) [39]. Finally, the library included a couple of biological and technical controls (~ 5%, see ref. [13] for details).

The second library [33, 35], comprising 100,000 variants, is composed of antigens derived from bacteriophages (40%), specifically selected for those infecting commensal gut bacteria, probiotic bacteria, and pathogenic bacteria. Moreover, this library includes allergens from six different databases (~ 30%) and all B cell antigens sourced from the Immune Epitope Database (IEDB) [40]. To avoid redundancy, antigen sequences that appeared in more than one database were included only once.

The process of antigen selection was based on a rational approach, prioritizing previously reported bacterial antigens and strains known to be recognized by the immune system. Additionally, uncharacterized antigens were given attention, particularly focusing on their functions, such as membrane-, surface-, motility-, and secreted proteins. Notably, the selection process did not consider peptide sequence length, necessitating peptide splitting within the limit of DNA oligonucleotide synthesis, which was set at 230 nucleotides. Detailed information regarding the precise composition of the libraries and the selection of antigens can be found elsewhere [13, 33, 35].

#### Antigen processing and library cloning

Antigens selected for library incorporation were segmented into peptides, each with a maximum length of 64 amino acids (aa) for the 244,000-variant library and 54 aa for the 100,000-variant library with an overlap of 20 aa between adjacent peptides. Aa sequences were reverse translated into DNA using the codon usage of *Escherichia coli* (of highly expressed proteins). During this process, restriction sites (*Eco*RI and *Hind*III) were excluded from cloning within the coding sequence. To achieve two unique barcodes at the 44/75-nucleotide (nt) location of each oligonucleotide in the coding sequence, coding was repeated when necessary [41]. Each barcode was designed to have a unique sequence at a specific Hamming distance, either three (44-nt) or five (75-nt) nucleotides, from all preceding sequences in the library. This design allowed for error correction during sequencing: single-read errors can be corrected when sequencing 44-nt barcodes and two-read errors can be corrected when sequencing 75-nt barcodes. Ultimately, the 44-nt sequencing approach was adopted, and a portion of the 5′-terminus was also sequenced to verify the presence of multiple inserts by confirming the match between 3′- and 5′-sequences. In cases where similar peptide sequences were identified, alternate codons were utilized based on the codon usage of *E. coli*, enabling effective discrimination. The sequence barcode was incorporated into the coding sequence, allowing the entire oligonucleotide to be used for peptide encoding, thereby making sequencing of the complete coding sequence unnecessary. Peptides < 64 aa were encoded by adding a random sequence after the stop codon, along with the addition of a SwaI restriction site (as a failsafe allowing for removal of short peptides through restriction enzyme digestion in case they overtook the signal, although this was not observed). Library amplification was performed by bringing together the *Eco*RI and *Hind*III restriction sites, antigen sequences, stop codons and annealing sequences. The oligomers for this amplification process were acquired as a pool of 230 oligomers for the 244,000-variant library (Agilent Technologies, Santa Clara, CA, USA) and a 200-oligomer pool for the 100,000-variant library (Twist Bioscience, San Francisco, CA, USA). The library amplification primers used were GATGCGCCGTGGGAATTCT (fwd) and GTCGGGTGGCAAGCTTTCA (rev). Antigen library cloning into the T7 phages was performed following the supplier’s instructions (Merck, T7Select 10–3 cloning kit, cat. no. 70550–3, Merck-Millipore, Burlington, MA, USA).

### Immunoprecipitation and next-generation sequencing

Bovine serum albumin (BSA, 150 μl, 30 g/L in Dulbecco’s phosphate-buffered saline) was used to block polymerase chain reaction (PCR) plates (used for bead transfer and washing) at 4 °C overnight incubation. Subsequently, BSA 2 g/L (final conc.) was added to the mixture containing diluted phages/antibodies for immunoprecipitation. The phage wash buffer consisted of 0.1 wt/vol% IPEGAL CA 630 (Sigma-Aldrich, cat. no. 13021). The phages (with 4000-fold coverage per library variants) and antibodies (3 μg of serum IgG antibodies quantified by ELISA) were mixed together following optimization procedures to determine the ideal amounts of phage and antibody for immunoprecipitation. Eventually, the 244,000-variant library was combined with the 200 nt 100,000-variant library pool at a 2:1 ratio. Deep well plates (96-format) were loaded with the phage-antibody mix while shaking on an overhead rotator at 4 °C. Protein A and -G magnetic beads were incubated with rotation at 4 °C overnight, and then 40 μL of this mixture was added to the wells at a 1:1 ratio following supplier’s instructions (Thermo-Fisher Scientific, cat. nos. 10008D and 10009D). Magnetic beads were subsequently added to the PCR plates using a Tecan Freedom Evo liquid-handling robot with filter tips. After a 4-h incubation, the beads were washed twice. Pooled Illumina amplicon sequencing was conducted using Q5 polymerase (New England Biolabs, cat. no. M0493L) for PCR amplification. The PCR primer pairs utilized were as follows: PCR1: tcgtcggcagcgtcagatgtgtataagagacagGTTACTCGAGTGCGGCCGCAAGC and gtctcgtgggctcggagatgtgtataagagacagATGCTCGGGGATCCGAATTC; PCR2: Illumina Nextera combinatorial dual index primers; PCR3 (of PCR2 pools): AATGATACGGCGACCACCGA and CAAGCAGAAGACGGCATACGA. PCR3 products were extracted from agarose gel and purified twice (1 × QIAquick gel extraction kit, 1 × QIAquick PCR purification kit; Qiagen cat. nos. 28704 and 28,104). Subsequently, PCR products were sequenced with custom primers: R1: ttactcgagtgcggccgcaagctttca and R2: tgtgtataagagacagatgctcggggatccgaattct (R1/R2, 44/31 nt). Finally, PCR products were paired-end sequenced using an Illumina NextSeq machine.

### Peptide sequence alignments and reference flagellins

The *blastp* application from BLAST® (v.2.10.1) was used to evaluate sequence similarity between antibody-bound flagellin peptides integrated in the PhIP-Seq antigen library and sample sequences of stimulator-, silent-, and evader-type flagellins that were used as references [12] (Table S1). Additionally, a group of 44 reference flagellins representing a more diverse spectrum of the three aforementioned representative flagellins was used to assess the classification of the antibody-bound flagellins (representing the set reported by Clasen et al. in their supporting Figure S4 [12], summarized in our Table S4). For the pairwise alignments with this larger group of reference sequences, pairwise2 of *biopython* (v. 1.83) was used to increase throughput. The extraction and annotation of N- and C-terminal domains were performed by mapping the corresponding domains in the reference flagellins using the Pfam protein domain references PF00669 (N-terminal domain) and PF00700 (C-terminal domain), similar to Clasen et al. [12]. For domain annotation, the respective domains of the antibody-bound flagellins were directly annotated using these Pfam references with the InterPro online tool followed by alignments to the reference domains as shown in the main figures and in Figures S1–S2 [42]. The reference domain sequences were also used for alignments with the full-length antibody-bound flagellins whereas the respective domain of the stimulator reference was used for all alignments (Figure S5A, C–E, G–I). For the N-termini of the flagellins with overrepresented antibody responses in CD, additional alignments were performed, as fewer sequences were mapped with the default references. In these cases, the silent reference was used in addition to domain detection optimization (Figure S5A, B, F); however, results remained largely unaffected when compared to the directly annotated domains.

Selected sequence alignments were performed to identify potentially shared motifs between multiple antibody-bound flagellin peptides using MEGA software (using the multiple sequence comparison by log-expectation [MUSCLE] algorithm in default settings) [43]. Visualization of these sequence alignments was performed using CLC Main Workbench 6 software.

Bacterial flagellins that induced greater binding/activation of TLR5 than FliC-PIM (indicating FliC of *S. typhimurium* with mutated key residues in the TLR5 motif reducing TLR5 binding) according to the data by Clasen et al. [12] were classified as ‘stimulators’, of which FliC from *S. typhimurium* served as a key example. Flagellins that showed weaker TLR5 binding and activation compared to FliC-PIM were classified as ‘evaders’, of which FlaA from *H. pylori* (*Hp*FlaA) served as a key example. Finally, ‘silent’ flagellins were defined as flagellins that showed strong TLR5 binding as evidenced by stronger interaction than FliC PIM but poor TLR5 activation capacity being worse than FliC PIM [12]. Here FlaB of *Roseburia hominis* (*Rh*FlaB) was selected as a key example since it was identified as the strongest silent flagellin by Clasen et al. [12]. The 44 flagellin references used for the alignments of category 2 (Fig. 4g, h, Figures S6, S7) were grouped according to these rules for classification.

### Statistical analysis

#### General statistical analyses

Demographic data were presented as mean ± standard deviation (SD) or proportions *n* with corresponding percentages (%). Continuously distributed data were compared between cohorts using Wilcoxon-Mann–Whitney tests while nominal variables were compared using chi-square tests, as appropriate. Spearman’s rank correlation coefficients were used to express associations of antibody responses between different cohorts. Comparisons of sequence alignment results between stimulator-, silent-, and evader-type flagellins were performed using Friedman tests, Wilcoxon’s matched-pairs signed rank tests, Kruskal–Wallis tests followed by Dunn’s post hoc tests with Bonferroni *P* value adjustment, or two-way analysis of variance (ANOVA) with Tukey’s post-hoc tests corrected for multiple comparisons. Two-tailed *P* values ≤ 0.05 were considered statistically significant. Statistical analyses were performed using the Python programming language (v.3.11, Python Software Foundation) using the *pandas* (v.1.2.3), *numpy* (v.1.20.0), *biopython* (v.1.8.1) and *scipy* (v.1.7.0) modules as well as GraphPad Prism (v.8.0.1, GraphPad Software, Inc.). Data visualizations were made using the *matplotlib* (v.3.4.1) and *seaborn* (v.0.11.1) packages in Python while schematic visualizations were created in BioRender.

#### PhIP-Seq data pre-processing

Raw DNA sequencing reads after immunoprecipitation were brought down to 1.25 million identifiable reads per sample, including reads within one error of the set of all possible barcodes of the two mixed phage libraries for which the paired end could match the identifiable peptide. In the situation that insufficient reads were obtained, at least 750,000 reads were enforced for data analysis. Peptide enrichment was determined by comparing the total number of reads for each peptide with that of the input read level (i.e., sequenced before immunoprecipitation). Input read levels per sample were considered to generate an output read level distribution that was fitted on a generalized Poisson distribution. Parameter estimation for this distribution was individually performed for each input read level in each individual sample. Subsequently, parameters were fitted to three distribution parameters for all samples following interpolation for each input read level, eventually generating scores. Corresponding *P* values were calculated and adjusted for multiple testing with Bonferroni correction, and adjusted *P* values (≤ 0.05) were considered statistically significant and defined as seropositivity. Fold changes in antibody-bound peptide enrichment were calculated as the number of reads after immunoprecipitation versus the number of reads before (input level) immunoprecipitation, which was calculated only for significantly enriched (i.e., seropositive) antibody-bound peptides and only with a minimum of 25 input reads per peptide. All remaining peptides were set to zero. Input sequencing of the phage libraries before immunoprecipitation was performed at > 100-fold coverage. Samples with < 200 significantly enriched antibody-bound peptides were excluded.

## Results

### High-throughput and high-resolution antibody repertoire profiling in CD and ME/CFS

We previously applied PhIP-Seq [44] to characterize antibody epitope repertoires in CD [10] and ME/CFS [11] against 344,000 rationally selected peptide antigens. PhIP-Seq allows for high-throughput and high-resolution detection of antibody responses against hundreds of thousands of antigens in parallel, making it one of the most comprehensive serological profiling technologies currently available [2]. Antigens are rationally selected, encoded as synthetic DNA (100,000 s of variants possible), and displayed on phages. After incubation with human blood samples, antibody reactivity is detected by immunoprecipitation and next-generation sequencing (Fig. 1a). Antigens represented in the phage libraries used in the CD [10] and ME/CFS [11] studies were derived from bacteria (including, pathogenic-, probiotic- and antibody-coated bacteria) and viruses, including antigens derived from metagenomics sequencing databases [13], as well as from virulence factors [39], allergens, phages [35], and B cell antigens from the IEDB [40].

We leveraged PhIP-Seq data from 256 patients with CD, an equally sized group of age- and sex-matched controls [33], 40 patients with ME/CFS, and an equal number of matched healthy controls assembled by the UK ME/CFS Biobank (UKMEB) [34]. Given the impact of age and sex on antibody repertoires [13], both patients with CD and ME/CFS were matched on these variables, showing no significant differences (Fig. 1b). We have previously reported on the potential use of these anti-flagellin antibody responses as biomarkers in both diseases. However, potential shared and distinctive aspects have not been studied, especially relating to the source of the flagellins: While our studies were under review, seminal work by Clasen et al. [12] reported on the existence of a third class of flagellins termed “silent” flagellins, beyond stimulators and evaders (Fig. 1c–d). However, it is unclear if these classes of flagellins are equally targeted in CD and ME/CFS, although this information may provide insights into their use as diagnostic markers and mechanistic differences between the two diseases.

### Anti-flagellin antibody responses in CD and ME/CFS show shared immune reactivity against the N-terminal domain

**Fig. 2:**
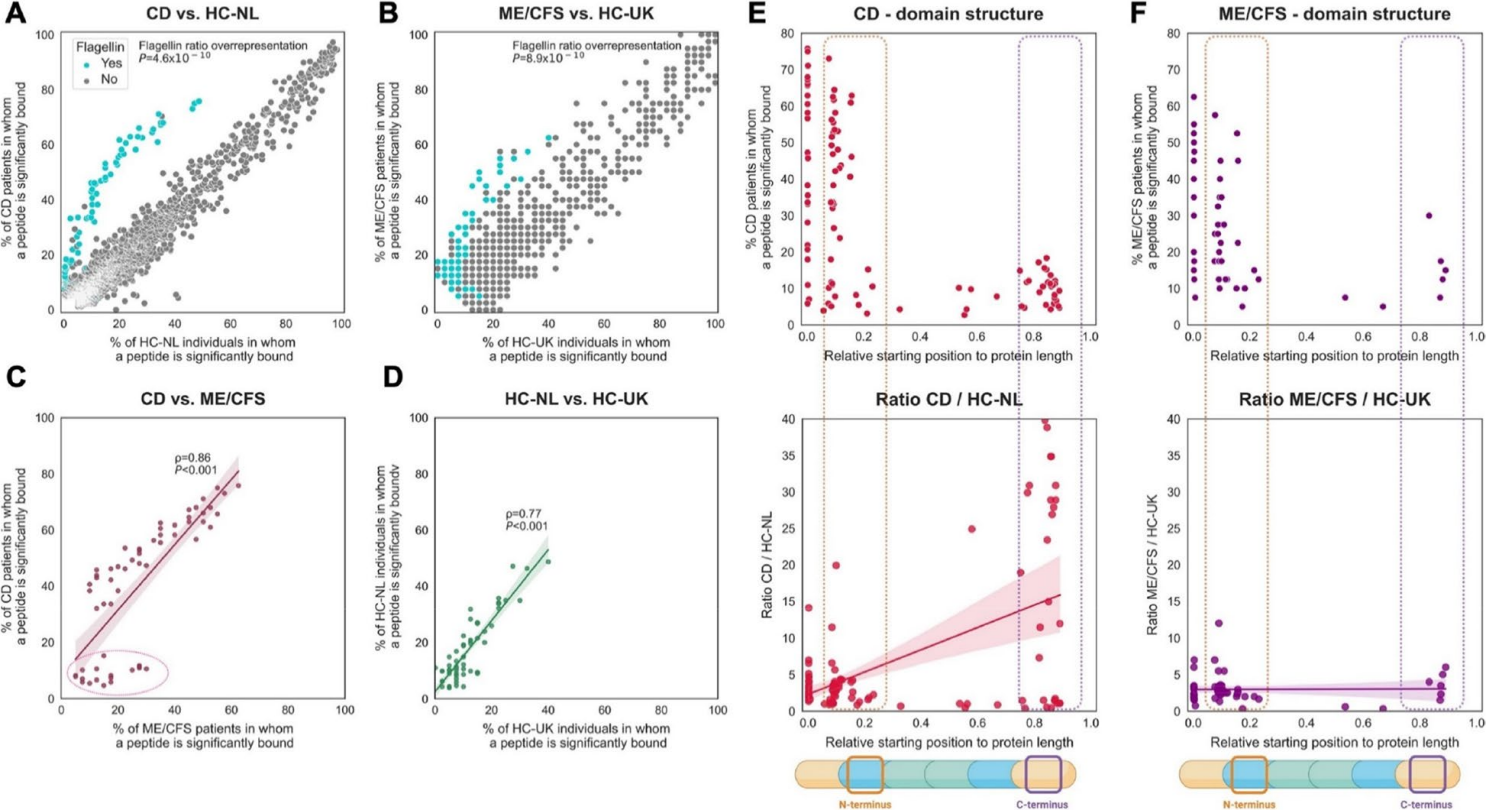
Anti-flagellin antibody responses are overrepresented in patients with CD and ME/CFS but show differences in epitope locations. **A** Anti-flagellin antibody responses are significantly overrepresented (Wilcoxon test on ratios for flagellin and non-flagellin peptides, *P* = 4.6 × 10^−10^) in patients with CD compared to healthy controls (HC-NL). **B** Similar as in **A**, patients with ME/CFS demonstrate an overrepresentation of anti-flagellin antibody responses (Wilcoxon test, *P* = 8.9 × 10^−^.^10^) compared to healthy controls (HC-UK). (**C**) Anti-flagellin antibody responses observed in patients with CD and ME/CFS are highly concordant (Spearman’s *ρ* = 0.86, *P* < 0.001) albeit a distinct set of antibody-bound peptides is found to be more prevalent in ME/CFS compared to CD (indicated by a pink dashed circle, Table S3, and see main text). **D** Anti-flagellin antibody responses likewise demonstrate a high concordance between both cohorts of matched healthy controls despite their different geographical origins. **E** Anti-flagellin antibody responses are directed against both the N-terminal (containing the TLR5 motif) and C-terminal (containing the allosteric binding site, which has only been identified in *Salmonella* FliC and is thus not necessarily a general feature of stimulator cD0s) regions of the flagellin peptide (upper panel). The relative starting positions of the antibody-bound peptides are marked (calculated as the position number of the starting amino acid divided by the total peptide length). Antibody-bound flagellin peptides that are highly overrepresented compared to matched controls (HC-NL), i.e., peptides that almost do not occur in healthy controls, are more commonly directed against the C-terminal region of the flagellin peptide (lower panel). **F** Similar to in **E**, anti-flagellin antibody responses are directed to both N-terminal and C-terminal regions of the flagellin peptide in patients with ME/CFS (upper panel). However, highly overrepresented antibody-bound flagellin peptides in ME/CFS do not exhibit a preferential epitope binding region (lower panel)

**Fig. 3:**
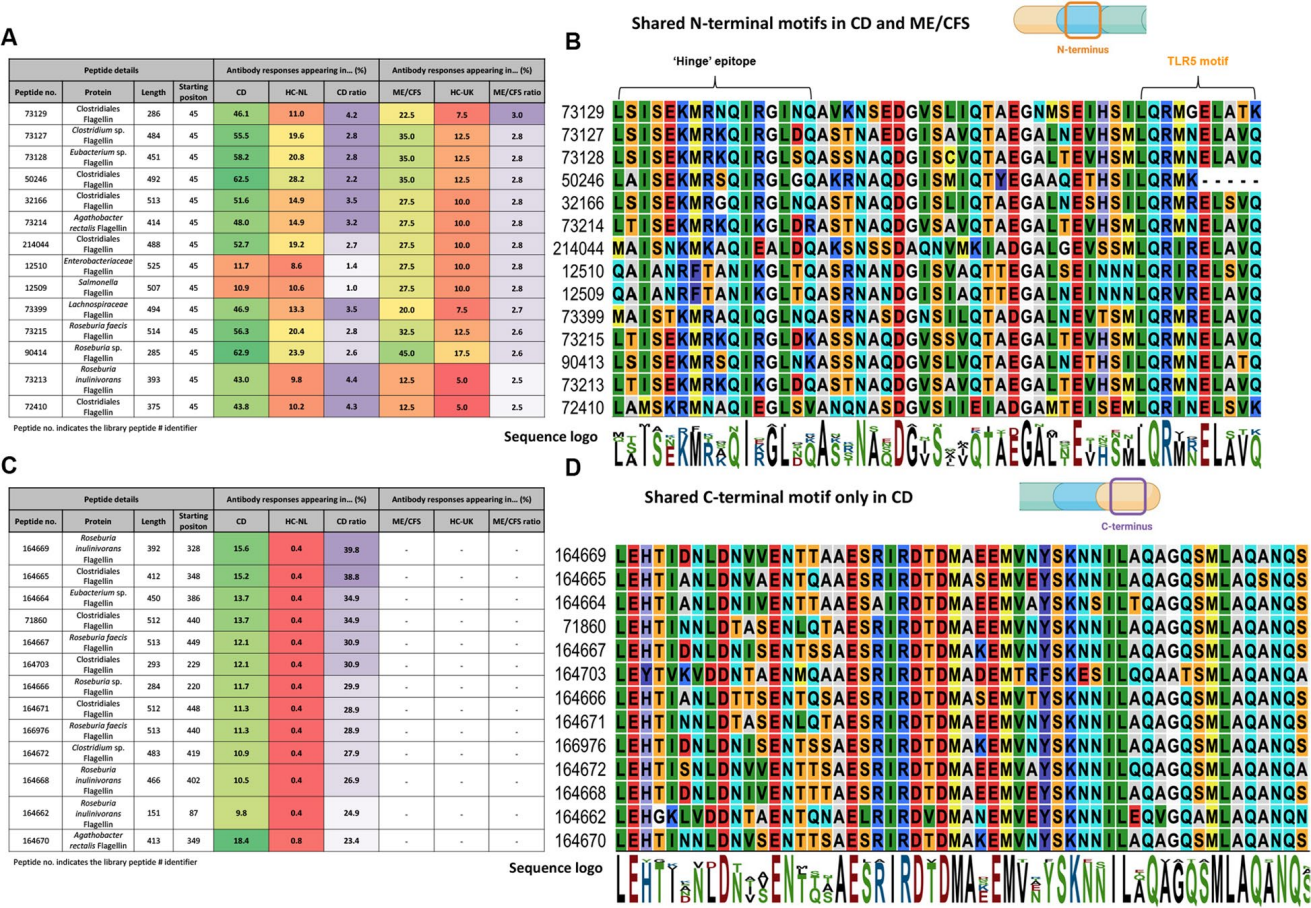
Antibody-bound bacterial flagellins overrepresented in patients with CD and ME/CFS share distinct N- and C-terminal sequence motifs. **A** Peptide details of antibody-bound flagellin peptides sharing N-terminal motifs. The table shows the library peptide # identifier, originating protein, peptide length, starting position of the peptide, and prevalence ratios of peptides in CD and ME/CFS cohorts. **B** Segment of sequence alignments of antibody-bound flagellin peptides overrepresented in both patients with CD and ME/CFS; the location of the hinge epitope (nD0-1) and TLR5 motif (nD1) are highlighted and show shared motifs putatively bound by antibody responses. See Figure S3a for the full alignment. **C** Peptide details of antibody-bound flagellin peptides sharing a C-terminal motif exclusively in patients with CD. The table shows the library peptide #, the original protein, peptide length, the starting position of the peptide, and prevalence ratios of peptides in CD. **D** Full sequence alignment of antibody-bound flagellin peptides that were > 25-fold overrepresented in patients with CD which all share a common C-terminal motif putatively bound by antibody responses. See Figure S3b for the full segment alignment and Figure S4 for the full alignment. Alignments were generated using MEGA software using the multiple sequence comparison by log-expectation (MUSCLE) algorithm in default settings (see “Materials and methods”)

High-resolution PhIP-Seq data [10, 11] provided a unique opportunity to study the characteristics, epitope binding, and structural (dis)similarity of anti-flagellin antibody responses. Anti-flagellin antibody responses against any type of flagellin were increased in patients with CD and ME/CFS compared to matched healthy controls (Wilcoxon tests, *P* = 4.6 × 10^−10^ and *P* = 8.9 × 10^−10^ for CD vs. HC-NL and ME/CFS vs. HC-UK, respectively) (Fig. 2a–b). The majority of these anti-flagellin antibody responses were targeted to bacterial taxa belonging to the order of Clostridiales and the family of *Lachnospiraceae*, including genera such as *Roseburia*, *Clostridium* and *Agathobacter*, but also to the family of *Eubacteriaceae* (genus *Eubacterium*) and pathogenic species such as *Salmonella*, *Legionella*, *Borrelia*, and *Burkholderia*. A full list of anti-flagellin antibody responses occurring in either CD, ME/CFS, or both can be found in Table S2. In addition, the key differences in bacterial taxa that contribute to binding via shared epitopes are visually represented in Fig. 3a, c, summarizing crucial information from the supplementary data.

Many of the observed anti-flagellin antibody responses were shared among the two diseases and demonstrated high concordance (Spearman’s *ρ* = 0.86, *P* < 0.001) (Fig. 2c). However, we identified a distinct set of anti-flagellin antibody responses to be more commonly present in patients with ME/CFS compared to patients with CD (Fig. 2c, lower left corner, Table S3). Remarkably, these antibody-bound flagellin peptides were almost exclusively derived from pathogenic bacterial species (rather than commensal bacteria like *Lachnospiraceae* to which the majority of anti-flagellin antibody responses were directed), including *Salmonella enterica*, *Escherichia coli* (O157:H7 strain), *Shigella flexneri*, *Shigella sonnei*, *Listeria monocytogenes*, *Legionella pneumophila*, and *Yersinia enterocolitica* (see Table S3). Although both cohorts of matched healthy controls differed in sample size and geographical origin (the Netherlands vs. UK), they still demonstrated a high concordance of anti-flagellin antibody responses (Spearman’s *ρ* = 0.77, *P* < 0.001) (Fig. 2d).

Subsequently, we aimed to determine to which regions of the flagellin protein the overrepresented anti-flagellin antibody responses were directed in patients with CD and ME/CFS. Directly comparing amino acid positions between flagellins is not possible, as the N- and C-terminal conserved domains are separated by a hypervariable region. To correct for this variable length, we calculated the relative starting position of each antibody-bound flagellin peptide by dividing the position number of the first amino acid of each peptide by the full length of the protein from which it was derived (i.e., the total number of amino acids, Fig. 2e, f). We also confirmed that the N-terminus and C-terminus consistently reside within the specified range of 30–50 amino acids, namely 91% of the N-terminal domains start in the first 5% of the sequence and 58% end between 25 and 34% of the sequence length. For the C-terminal domains, 87% start between 75 and 86% while 98% end between 99 and 100% of the complete length (Supplementary Figure S7a).

Comparing frequencies of anti-flagellin antibody responses in patients with CD against the relative starting position of peptides, we observed antibody responses directed to both the N- and C-terminal regions of the flagellin protein (Figs. 2e and 3, Figure S3, Figure S4). Although most anti-flagellin antibody responses were directed to the N-terminal region (Fig. 2e, upper panel, Fig. 3a, b), there was also substantial binding to the C-terminal region. The latter binding against the C-terminal domain largely consisted of antibodies that were very highly overrepresented (> 15-fold) in patients with CD compared to their matched controls (HC-NL) (Fig. 2e, lower panel, Fig. 3c,d). These anti-flagellin antibody responses against the C-terminal domain in CD were exclusively directed towards flagellins from commensal bacteria including *Lachnospiraceae* bacteria like *Roseburia*, *Clostridium*, and *Butyrivibrio*, as well as flagellins from *Eubacterium* (Table S2).

Similar to patients with CD, anti-flagellin antibody responses were also directed to both N- and C-terminal regions in patients with ME/CFS, but only very few to the C-terminal region (Fig. 2f, upper panel). When comparing ratios of anti-flagellin antibody responses in patients with ME/CFS vs. matched controls (HC-UK), there was no apparent preferentially targeted region of the flagellin protein (Fig. 2f, lower panel).

### Antibody-bound flagellins overrepresented in CD and ME/CFS resemble stimulator and silent flagellins but not evader flagellins

**Fig. 4:**
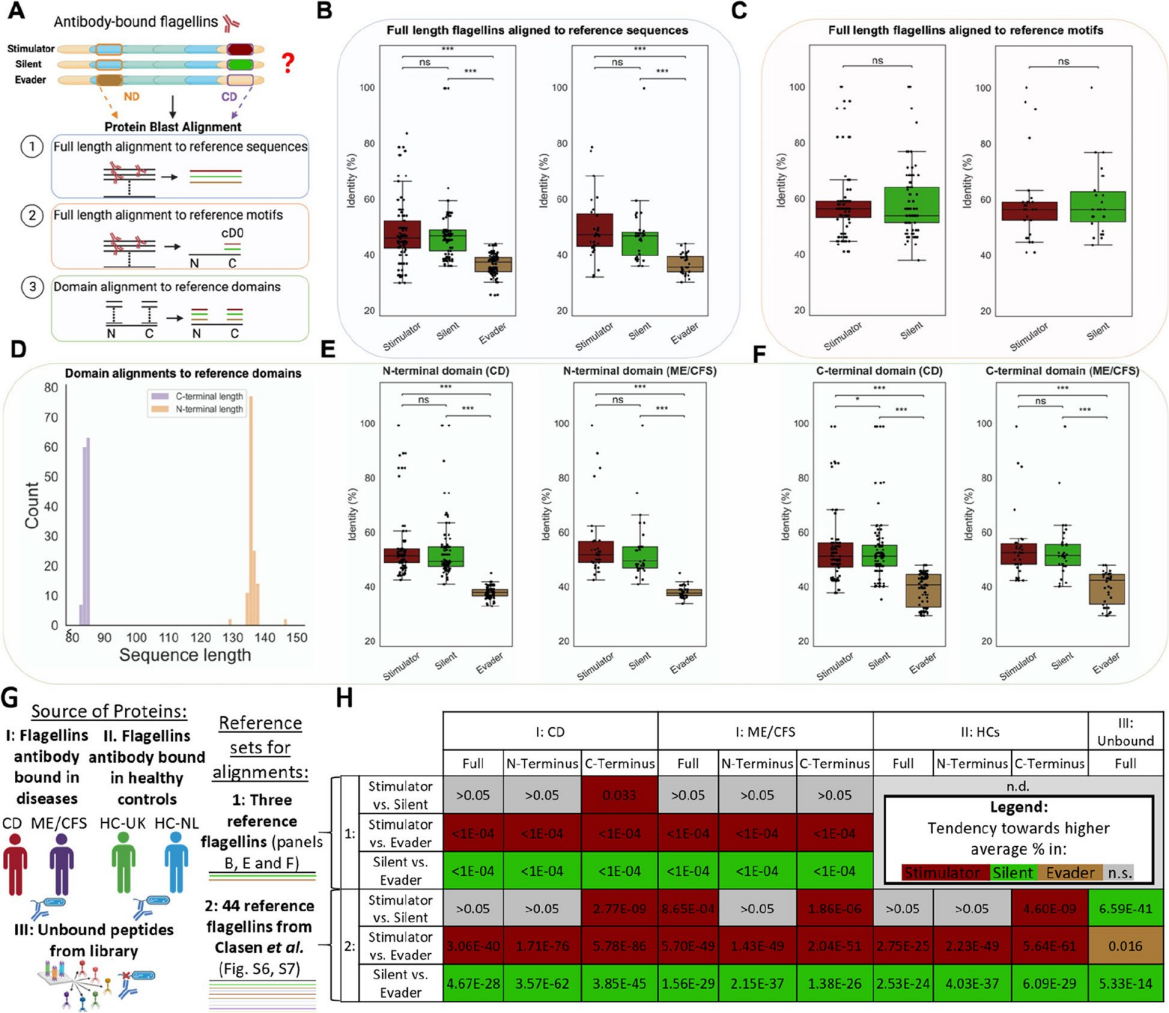
Sequences of antibody-bound flagellins overrepresented in CD and ME/CFS resemble both stimulator- and silent flagellins while C-terminal antibody-bound flagellin sequences exhibit a higher resemblance to stimulator flagellins in CD. **A** Overview of the three main alignment strategies (see Figure S2, S6, and S7 for alternative alignments) that were used to compare the overrepresented antibody-bound flagellins against reference sequences of stimulator-, silent- and evader-type flagellins derived from Clasen et al. [12]. **B** Boxplots showing the degree of sequence identity (%) for full-length antibody-bound flagellin peptides towards reference sequences of stimulator-, silent-, and evader-type flagellin peptides (left panel: CD; right panel: ME/CFS). **C** Boxplots demonstrating the degree of sequence identity (%) between full-length antibody-bound flagellin peptide sequences and reference motifs for the C-terminal D0 domain (containing the allosteric binding site) (left panel: CD; right panel: ME/CFS). **D** Histogram showing the distributions of sequence lengths of extracted and annotated N- and C-terminal domains of antibody-bound flagellin peptides. **E** Boxplots demonstrating the degree of sequence identity (%) between N-terminal domains of antibody-bound flagellin peptides and N-terminal domains of reference sequences for stimulator-, silent-, and evader-type flagellin in patients with CD and ME/CFS. **F** Boxplots demonstrating the degree of sequence identity (%) between C-terminal domains of antibody-bound flagellin peptides and C-terminal domains of reference sequences for stimulator-, silent-, and evader-type flagellin in patients with CD and ME/CFS. **G** Overview of the performed alignments and the used references. **H** Table listing the *P* values of all alignment comparisons. Due to the limitations of GraphPad Prism, *P* values smaller than 0.0001 could not be displayed for the alignments of group 1. *P* values for comparison of sequence identity (%) in panels **B**, **C**, **E**, and **F** were derived from Friedman tests. For panel H, Kruskal–Wallis tests followed by Dunn’s post hoc tests with Bonferroni *P* value adjustment were used. ^*^P < 0.05; ^***^P < 0.001; ns, non-significant

Subsequently, we aimed to compare the sequence similarity of antibody-bound flagellin peptides found to be overrepresented in patients with CD and ME/CFS by identifying shared motifs across peptide sequences (Fig. 3) and by comparing them against reference sequences of stimulator-, silent-, and evader-type flagellins (Fig. 4, Figure S1, Table S1). Reference sequences and their definitions (see “Materials and methods”) were retrieved from Clasen et al. [12].

Using BLAST® for alignments, we assessed the sequence similarity between PhIP-Seq-derived flagellin peptide sequences that were overrepresented in CD and ME/CFS (Table S2) with the three reference sequences for stimulator- (FliC), silent- (*Rh*FlaB), and evader-type (*Hp*FlaA) flagellins (Fig. 4a). When aligning full-length antibody-bound flagellin proteins with the entire length of the reference sequences, significantly higher sequence similarity was observed to stimulator- and silent flagellins compared to evader-type flagellin in both CD and ME/CFS (Fig. 4b). Similar results were obtained when aligning only the antibody-bound peptides (instead of the full-length proteins) (Figure S2a–d) or when specifically comparing antibody-bound flagellins that were highly (> 2.5 fold) overrepresented in either CD or ME/CFS (Figure S2e–h). Subsequently, sequence similarity was compared between full-length antibody-bound flagellin sequences and the C-terminal (cD0) reference motifs (also from Clasen et al. [12]) which contain an active allosteric binding site for TLR5 in stimulator but not in silent flagellins. This analysis revealed no significant difference in similarity to either stimulator- or silent-type flagellins in CD or ME/CFS (Fig. 4c). However, in these alignments, similarities to other regions and issues of the algorithms with aligning large differences in length may bias outcomes.

Given this issue and the distinct binding to N-terminal and C-terminal domains of flagellin sequences among the three classes of flagellins (as well as the observed relationships between flagellin peptide prevalence and relative starting positions shown in Fig. 2e–f), we further aimed to evaluate N- and C-terminal domain-specific sequence similarity between antibody-bound flagellins and the reference sequences. To this end, the N- and C-terminal domains were extracted and annotated for all antibody-bound flagellins overrepresented in CD and ME/CFS and for the reference sequences, which on average spanned ~ 130–140 aa for N-terminal and ~ 80–90 aa for C-terminal domains (Fig. 4d). Sequence alignment of N-terminal domains revealed a high resemblance to stimulator- and silent-type flagellins in both CD and ME/CFS but not to evader-type flagellins (Fig. 4e), in line with results of the full-length alignments (Fig. 4a). A summary of antibody-bound peptides sharing a common N-terminal motif is shown in Fig. 3. Two distinct regions (the ‘hinge’ epitope at nD0-1 and the TLR5 motif at nD1) are highlighted (Fig. 3a) which are both parts of conserved N-terminal motifs, that appeared in the 64 amino acid peptides bound by antibodies. Alignment of the C-terminal domains revealed a similar resemblance to stimulator- and silent flagellins for ME/CFS (Fig. 4f, right panel), but in patients with CD, a significantly higher resemblance to stimulator flagellin was observed compared to silent flagellin (Fig. 4f, left panel). In fact, antibody-bound flagellin peptides that were highly (> 20-fold) overrepresented in patients with CD compared to controls (see also Fig. 2e) demonstrated the most strongly conserved motif at the C-terminal end (Fig. 3c–d). Similar findings as presented in Fig. 4 were obtained when e-values were compared instead of the identity percentages after performing sequence alignment comparisons (Figure S1, Figure S5).

To further corroborate these results, we extended the analysis shown in Fig. 4a–f (performed in CD/CFS and ME/CFS patients with three reference flagellins) also to healthy controls, unbound flagellins from the library as well as a larger set of 44 flagellins (tested by Clasen et al. [12]) as summarized schematically in Fig. 4g. In general, alignments with the larger set of 44 flagellins confirmed the results from the three reference flagellins (Fig. 4h, Figure S6–S7): Antibody bound flagellins in CD and ME/CFS patients were significantly more similar to stimulator- and silent flagellins than to evaders etc. (Fig. 4h). A difference was observed in the C-terminal domain for CD vs. ME/CFS, which was significant in both cases. However, the difference in CD was ~ 1000-fold more significant (*P* = 2.8 × 10^−9^ vs. *P* = 1.9 × 10^−6^).

Regarding differences between CD, ME/CFS patients vs. healthy controls, from the data shown in Fig. 3 (and Table S2), it seems that there is no strong difference in the types of flagellins bound in CD/CFS patients vs. healthy controls. The same flagellins appear also in healthy individuals, however, antibody binding occurs at lower frequencies than in CD/CFS (see panels c vs. d of Fig. 2). These overlaps are illustrated in Venn diagrams in Figure S6a. Indeed, when repeating the analysis of the flagellins bound in healthy controls, we found similar trends as in CD and ME/CFS, supporting that these differences are rather due to the frequency of antibody binding (Fig. 4h, Figures S6–S7, and discussion therein).

Lastly, we also checked whether the selection of flagellins in our antigen library could bias our results. Therefore, we ran a similar analysis on all flagellins included in our antigen library, but not bound by antibodies in CD/CFS patients or healthy controls. Running the alignments on this group (referred to as “unbound”), showed a different pattern from antibody-bound flagellins (Fig. 4h, Figures S6–S7, and discussion therein). Namely, in this case, there was no significant difference between stimulator- vs. evader-type flagellins, although there was a significant difference between stimulator vs. silent flagellins. Hence, the findings related to antibody-bound flagellins not merely mimic the library content but even show effects in opposite directions.

## Discussion

Altogether, our work shows that elevated systemic antibody responses against bacterial *Lachnospiraceae* flagellins in patients with CD and ME/CFS target primarily a shared N-terminal region, while C-terminal antibody responses appear more CD-specific. When classifying overrepresented antibody-bound flagellin peptides into stimulator-, silent-, and evader-type flagellins, sequences of antibody-bound flagellins mostly resembled stimulator- and silent-type flagellins, a phenomenon consistently observed in both CD and ME/CFS. While the antibody-bound sequences in the N-terminal flagellin domain (nD0-1) were similar across CD and ME/CFS patient groups, C-terminal antibody-bound flagellin sequences in patients with CD displayed a higher degree of resemblance to stimulator- than to silent-type flagellins. Of note, a distinct subset of antibody-bound flagellins was exclusively identified in a subset (10–20%) of patients with CD and characterized by its strong overrepresentation (exceeding 20-fold), underscoring its potential significance in distinguishing pathophysiologic subtypes of CD, and setting it apart from ME/CFS.

### Implications of the antibody responses detected

The *Lachnospiraceae* bacterial family, characterized by mostly anaerobic bacteria that produce short-chain fatty acids (SCFAs) known for their anti-inflammatory and intestinal barrier-protective properties, has been reported to decrease in prevalence among patients with CD [45]. Numerous flagellated members of the *Lachnospiraceae* family act as commensal symbionts, rendering their immunogenicity, although widely observed [7, 9, 46–48], somewhat puzzling. Nonetheless, the potent immunogenic attributes of flagellins suggest a potential role in facilitating bacterial passage through the mucus barrier covering the intestinal epithelium [49]. A plausible hypothesis emerges wherein antibodies targeting flagellated *Lachnospiraceae* could potentially contribute to the reduced abundance of these microbes already in the early stages of IBD [50]. However, to study these aspects, also metagenomic sequencing of stool samples would need to be performed (see “Limitations of this study” section for details and a discussion of relevant literature references). Recent findings presented in a preprint by Zhao et al. propose the intriguing notion that this phenomenon might be rooted in a homeostatic immune response initiated during infancy that ordinarily diminishes into adulthood [47]. This, in turn, paves the way for contemplating the specificity of the immune response characterized by anti-flagellin antibodies in the context of CD, distinct from their absence in UC. This discrepancy underscores the fundamental divergence in the pathophysiological underpinnings of these diseases. Such variations could potentially originate from CD-specific immune responses, which may include aberrant Th1-driven immunity or certain genetic predispositions [2].

### Role of different flagellin types

The seminal study by Clasen et al. [12] has given a new perspective to these observations: Anti-flagellin antibody responses may exert different effects, depending on which functional class (stimulator, silent, or evader) is bound. Within this framework, we present evidence of antibody binding to the N-terminal domain of both stimulator- and silent-type flagellins, an interaction that seems absent with evader-type flagellins. This engagement of antibodies may potentially impede TLR5 activation in patients with CD and ME/CFS. It is conceivable that if this blockade of flagellin-TLR5 interaction mirrors the consequences of mutations in critical residues of the TLR5 motif akin to evader-type flagellins, this could lead to an immune activation of commensal bacteria (such as *Lachnospiraceae*) similar to an immune activation by default elicited by pathogenic evaders. This scenario might consequently contribute to the dysregulation of innate immunity in both diseases.

Furthermore, elevated antibody binding to the C-terminal D0 domain, encompassing the flagellin TLR5 allosteric binding site, observed for stimulator-type flagellins in CD may also alter TLR5 activation and potentially transform these stimulator-type flagellins into silent-type counterparts (or in cases of silent-type flagellins, further diminishing their likelihood of activating TLR5). The C-terminal domain of flagellin is recognized by the intracellular sensor NLRC4 [12, 51–53], pointing towards another mode of innate immune activation potentially involved.

Beyond the cD0 domain, interestingly, Zhao et al. [47] observed elevated systemic antibody responses against a prominent B cell peptide epitope residing within the ‘hinge region’ of the N-terminal domain (nD0-1, p25-59) of *Lachnospiraceae* flagellins in CD. However, antibody binding to the TLR5 motif region (nD1, p74-102) was less frequently observed. This observation contrasts with a prior study in which antibody-mediated neutralization of flagellin-TLR5 activation was observed, prompted by antibodies targeting both the conserved D0/D1 region and the flagellin’s hypervariable regions [54]. Yet it remains unclear what distinctive roles would be played by these antibody responses in neutralizing TLR5 activation and to what extent each of these responses contributes to the pathogenesis of the diseases at hand. This intricate interplay warrants further investigation to unravel the nuanced mechanisms underlying the immunopathogenesis of CD and ME/CFS.

### Limitations of this study

Our study has several limitations. Among these is the absence of experimental validation to substantiate the findings stemming from the performed sequence alignments, including the full-length comparisons against reference sequences of stimulator-, silent- and evader-type flagellins, comparisons involving the cD0 reference motifs, and comparisons specific to the N- and C-terminal-specific domains. Ideally, antibody binding against these regions should be tested in orthogonal assays, as well as TLR5 binding studies (by allosteric and TLR5 motif-based pathways). This work would require monoclonal antibodies (mAbs) against the respective regions of the flagellins. Obtaining such mAbs would require the isolation of B cells and the subsequent purification of anti-flagellin-specific antibodies from patients (using patient sera would be insufficient as these encompass polyclonal rather than monoclonal responses). As we did not collect PBMCs for our cohorts (required for B cell isolation and mAbs production), executing this validation poses significant challenges. Using our remaining blood samples, the inherent diversity in reactivity will make it unfeasible to precisely pinpoint the exact antigenic epitopes to which these antibodies bind.

Animal models could present an interesting approach to validate these findings (by vaccinating with different flagellins) however would introduce additional layers of complexity: there are potential dissimilarities between the human- and mouse immune systems, *e.g.* differences in major histocompatibility complex (MHC) structures and CD4^+^-T-helper cell recognition [55] both of which are required for B cell activation and the subsequent production of these antibodies. In fact, the antibody responses elicited in mice via immunization with peptides from human pathogens might even manifest as a shift toward distinct proteins as has been shown previously [13].

Additional limitations inherent to this study are associated with the utilization of the PhIP-Seq technology. While this approach has established comprehensive insights into anti-flagellin antibody responses at the granularity of peptide-level resolution (wherein multiple overlapping peptides per distinct protein were considered), certain epitopes such as those featuring misfolded, conformational-, or non-protein structures, as well as posttranslational modifications, are typically not detected. Despite this major constraint, it should be noted that the PhIP-Seq approach surpasses conventional antibody profiling technologies (e.g., peptide arrays) by a substantial magnitude, even if only a mere 10% of the potential antigenic space were to be detected [11]. Another potential approach to validate findings could be to work with live bacteria rather than peptide antigens displayed on phages. However, a key issue would emerge in this theoretical case, which is that bacteria express several flagellins at once [12], making it hard to disentangle flagellin-specific effects. Instead, one would need to create specific knock-outs or induce overexpression of a strain in non-flagellated bacteria.

It would also be highly relevant to compare the antibody responses measured to the metagenomic composition of the respective individuals. However, for the ME/CFS patients, we lack unfortunately MGS (as well as stool samples), precluding the option to perform this analysis. However, two recent studies did investigate the microbiome compositions of ME/CFS patients [56, 57]. Interestingly, they reported reduced abundances of *Lachnospiraceae* bacteria as well as reduced levels of short-chain fatty acids (SCFAs) in ME/CFS patients compared to healthy controls (*Lachnospiraceae* are SCFA-producers). Hence, it is tempting to speculate whether antibodies against these bacteria could reduce their abundance and metabolic contribution. However, studying the direction and causality in this setting will require future studies and assembling new cohorts.

More generally, we have previously studied the connection between the microbiome composition (inferred from metagenomics sequencing) and antibody repertoires as determined by PhIP-Seq, in both healthy individuals [13] and patients with CD [10]. As discussed in these works, we did not find a strong concordance which may be due to different reasons (detection limits of the methods, technical aspects such as library size and sequencing depth, bacteria generating long-lasting antibodies detected by PhIP-Seq may have already been eradicated by the immune system and not be detectable anymore, location of serum antibodies vs. gut microbiota etc. [10, 13]).

Finally, our findings might potentially be influenced by specific features of the cohorts of patients with CD and ME/CFS that were examined. Variations in detected antibody responses might be attributed to other factors, including unseen genetic- and environmental factors. However, it is worth highlighting that efforts were made to ascertain comparable age- and sex distributions across all cohorts. While our cohorts were not significantly different for age and sex, the ME/CFS cohort is somewhat younger and contained slightly more female participants. However, despite differences in sample size and geographical origin, it is also important to note that antibody responses observed within both healthy control cohorts exhibited a substantial correlation, suggesting that the disease-specific signals outweigh any potential cohort effects.

### Concluding statement

Ultimately, despite all these limitations, the results presented in this study highlight the diagnostic potential of anti-flagellin antibody responses in both CD and ME/CFS and provide a basis for further mechanistic investigations of flagellin-TLR5 interactions and their impact on the delicate equilibrium between innate and adaptive immunity. Our findings raise questions about the potentially pathogenic role of anti-flagellin antibody responses in both CD and ME/CFS. One prominent example revolves around the potential pathogenic significance of antibody responses directed towards the C-terminal region of primarily stimulator-type flagellins, a phenomenon predominantly exclusive to a specific subset of patients with CD. Additionally, the exploration of mucosal versus systemic domain-specific anti-flagellin antibody responses, targeting either the N- or C-terminal regions, presents an avenue to examine whether mucosal IgA-mediated responses might contribute to curtailing systemic immune activation, potentially presenting a mode of immune regulation.

Overall, the main question that remains unanswered relates to what precise sequence of events underpins the emergence of anti-flagellin antibody responses. Do these responses instigate primary pathogenic cascades in CD or ME/CFS—such as impeding TLR5 activation, thereby disrupting innate immune pathways—or do they merely manifest as collateral phenomena resulting from compromised intestinal barrier integrity or mucosal injury? Moreover, there are perspectives for manipulating anti-flagellin antibody responses for therapeutic purposes. Earlier research has already demonstrated the feasibility of targeted elimination of CD4^+^ T-memory cells responsive to flagellin through immunotherapy guided by flagellin peptides [58]. This intervention showed the capacity to prevent colitis in murine models through a combination of cell activation and metabolic checkpoint inhibition. These developments extend into the development and evaluation of immunotherapeutic strategies targeted to flagellin, potentially offering therapeutic avenues for patients with CD and/or ME/CFS.

### Supplementary Information

Supplementary Material 1: Supplemental Table S1. Reference sequences of stimulator-, silent-, and evader-type flagellins derived from Clasen *et al*. [22]. Supplemental Table S2. Full list of antibody-bound flagellin peptides occurring in either CD, ME/CFS, or both (prevalence>5% in either cohort). Supplemental Table S3. List of antibody-bound flagellin peptides shared in both CD and ME/CFS cohorts with highlighted antibody-bound peptides occurring more often in ME/CFS (purple) than in CD (red) (prevalence >5% in either cohort). Supplemental Table S4. List of 44 flagellin references and their N- and C-terminal domains derived from Clasen *et al*. [22] (obtained by searching for UniProt numbers listed in their supporting Fig. S4).

Supplementary Material 2: Supplemental Figure S1. Sequences of antibody-bound flagellins overrepresented in CD and ME/CFS resemble both stimulator- and silent flagellins while C-terminal antibody-bound flagellin sequences exhibit higher resemblance to stimulator flagellins in CD. (A-B) Boxplots showing the degree of statistical significance (e-values) for full-length antibody-bound flagellin peptides towards reference sequences of stimulator-, silent-, and evader-type flagellin peptides (panel A: CD; panel B: ME/CFS). (C-D) Boxplots demonstrating the degree of statistical significance (e-values) between full-length antibody-bound flagellin peptide sequences and reference motifs for the C-terminal D0 domain (containing the allosteric binding site) (panel C: CD; panel D: ME/CFS). (E-F) Boxplots demonstrating the degree of significance (e-values) between N-terminal domains of antibody-bound flagellin peptides and N-terminal domains of reference sequences for stimulator-, silent-, and evader-type flagellin in patients with CD (left panel) and ME/CFS (right panel). (G-H) Boxplots demonstrating the degree of significance (e-values) between C-terminal domains of antibody-bound flagellin peptides and C-terminal domains of reference sequences for stimulator-, silent-, and evader-type flagellin in patients with CD (left panel) and ME/CFS (right panel). Supplemental Figure S2. Peptide sequences of antibody-bound flagellins overrepresented in CD and ME/CFS resemble full-length stimulator- and silent flagellins and highly (>2.5 fold) overrepresented full-length antibody-bound flagellins overrepresented in CD and ME/CFS resemble both stimulator- and silent flagellins. (A-B) Boxplots showing the degree of sequence similarity (identity%, left panel) and statistical significance (e-values, right panel) for peptide-length antibody-bound flagellins in CD towards reference sequences of stimulator-, silent-, and evader-type flagellin peptides. (C-D) Boxplots showing the degree of sequence similarity (identity %, left panel) and statistical significance (e-values, right panel) for peptide-length antibody-bound flagellins in ME/CFS towards full-length reference sequences of stimulator-, silent-, and evader-type flagellin peptides. (E-F) Boxplots showing the degree of sequence similarity (identity %, left panel) and statistical significance (e-values, right panel) for highly (>2.5 fold) overrepresented full-length antibody-bound flagellins in CD towards reference sequences of stimulator-, silent-, and evader-type flagellin peptides. (G-H) Boxplots showing the degree of sequence similarity (identity %, left panel) and statistical significance (e-values, right panel) for highly (>2.5 fold) overrepresented full-length antibody-bound flagellins in ME/CFS towards full-length reference sequences of stimulator-, silent-, and evader-type flagellin peptides. Supplementary Figure S3. Full segment alignments of antibody-bound bacterial flagellins overrepresented in patients with CD and ME/CFS sharing distinct N- and C-terminal sequence motifs. (A) Full alignment of subset of antibody-bound flagellin peptides overrepresented in both patients with CD and ME/CFS; the location of the hinge epitope (nD0-1) and TLR5 motif (nD1) are highlighted and show shared motifs putatively bound by antibody responses. (B) Full alignment of antibody-bound flagellin peptides that were>25 fold overrepresented in patients with CD which all share a common C-terminal motif putatively bound by antibody responses. Alignments were generated using MEGA software using the multiple sequence comparison by log-expectation (MUSCLE) algorithm in default settings (see Methods). Supplementary Figure S4. Full alignments of antibody-bound bacterial flagellins overrepresented in patients with CD showing both N- and C-terminal sequence motifs. Shown is the full alignment of antibody-bound flagellin peptides overrepresented in patients with CD in which both the N-terminal motifs shared with patients with ME/CFS is observed (albeit in a different order, lower red rectangle) as well as the CD-specific C-terminal motif (upper red rectangle). Alignments were generated using MEGA software using the multiple sequence comparison by log-expectation (MUSCLE) algorithm in default settings (see Methods). Supplementary Figure S5. Sequences of antibody-bound flagellins overrepresented in CD and ME/CFS resemble both stimulator- and silent flagellins also using alternative domains for alignments. (A) Histogram showing the distributions of sequence lengths of extracted and annotated N- and C-terminal domains of antibody-bound flagellin peptides when using the silent domain rather than the stimulator one as reference for the N-termini (see Methods). (B-C) Boxplots demonstrating the degree of sequence identity (%) between N-terminal domains of antibody-bound flagellin peptides and N-terminal domains of reference sequences for stimulator-, silent-, and evader-type flagellin in patients with CD and ME/CFS, yielding similar results as shown in Fig. 4, Fig. S1, and Fig. S2, demonstrating that the selection of the reference domain for alignments does not strongly impact outcomes. (D-E) Boxplots demonstrating the degree of sequence identity (%) between C-terminal domains of antibody-bound flagellin peptides and C-terminal domains of reference sequences for stimulator-, silent-, and evader-type flagellin in patients with CD and ME/CFS. (F-G) Boxplots demonstrating the degree of significance (e-values) between N-terminal domains of antibody-bound flagellin peptides and N-terminal domains of reference sequences for stimulator-, silent-, and evader-type flagellin in patients with CD (left panel) and ME/CFS (right panel). (H-I) Boxplots demonstrating the degree of significance (e-values) between C-terminal domains of antibody-bound flagellin peptides and C-terminal domains of reference sequences for stimulator-, silent-, and evader-type flagellin in patients with CD (left panel) and ME/CFS (right panel). Supplementary Figure S6. In contrast to unbound flagellins, the antibody bound flagellins in disease- and healthy control cohorts overlap in occurrence and resemble the stimulator and silent subgroups of 44 reference flagellins. (A) Venn diagrams illustrating overlaps of the antibody bound flagellins in CD, ME/CFS, HC-NL and HC-UK. (B) Bar plot showing the degree of sequence identity (%) for full length antibody-bound flagellin peptides in CD towards each of the 44 reference sequences (Table S4). (C) Boxplots showing the summarized degree of sequence identity (%) for full length antibody-bound flagellin peptides in CD, ME/CFS, and the HCs as well as unbound flagellins towards the 44 reference sequences (Table S4). *P*-values for comparison of sequence identity (%) were derived from Kruskal-Wallis tests followed by Dunn’s post hoc tests with Bonferroni *P*-value adjustment. Supplementary Figure S7. N- and C-terminal domain-sequences of antibody-bound flagellins overrepresented in CD and ME/CFS as well as domain sequences of flagellins antibody-bound in HCs resemble both stimulator- and silent flagellin group of 44 references while C-terminal antibody-bound flagellin sequences exhibit higher resemblance to stimulator flagellins in CD, ME/CFS and the HCs. (A) Histogram showing the distributions of the relative start and end positions of the N- and C-terminal domains of antibody-bound flagellins in CD. The brackets describe the ratio of all domain sequences in contained the marked bins. (B) Boxplots demonstrating the summarized degree of sequence identity (%) between N- and C-terminal domains of antibody-bound flagellin peptides and N- and C-terminal domains of 44 reference sequences (Table S4) in patients with CD. (C) Boxplots demonstrating the summarized degree of sequence identity (%) between N- and C-terminal domains of antibody-bound flagellin peptides and N- and C-terminal domains of 44 reference sequences (Table S4). Al in patients with ME/CFS. (D) Boxplots demonstrating the summarized degree of sequence identity (%) between N- and C-terminal domains of antibody-bound flagellin peptides and N- and C-terminal domains of 44 reference sequences (Table S4) in the healthy control cohorts.* P*-values for comparison of sequence identity (%) were derived from Kruskal-Wallis tests followed by Dunn’s post hoc tests with Bonferroni *P*-value adjustment.

## Data Availability

The datasets used and/or analyzed for the current study are directly included in the manuscript/supporting files or available via the links provided below. PhIP-Seq datasets from CD patients and matched population controls are available under the accession numbers EGAD00001010118 and EGAS00001006999 (see [10] and [33]). All data from the ME/CFS cohort are incorporated in a previous publication [11] and available via https://github.com/kalkairis/PhageIPSeq_CFS. Supplementary tables and custom code used for analyses in this study are available in a GitHub repository via the following link: https://github.com/abourgonje/Flagellin_Project and will be made publicly available from the date of publication onwards.
