# Supplementary material for: Systemic antibody responses against gut microbiota flagellins implicate shared and divergent immune reactivity in Crohn’s disease and chronic fatigue syndrome
Source: Microbiome. 2024 Jul 29;12:141. doi: 10.1186/s40168-024-01858-1 (PMC11285207; doi:10.1186/s40168-024-01858-1)
Supplement: Supplementary file 3 — Supplementary Material 2: Supplemental Figure S1. Sequences of antibody-bound flagellins overrepresented in CD and ME/CFS resemble both stimulator- and silent flagellins while C-terminal antibody-bound flagellin sequences exhibit higher resemblance to stimulator flagellins in CD. (A-B) Boxplots showing the degree of statistical significance (e-values) for full-length antibody-bound flagellin peptides towards reference sequences of stimulator-, silent-, and evader-type flagellin peptides (panel A: CD; panel B: ME/CFS). (C-D) Boxplots demonstrating the degree of statistical significance (e-values) between full-length antibody-bound flagellin peptide sequences and reference motifs for the C-terminal D0 domain (containing the allosteric binding site) (panel C: CD; panel D: ME/CFS). (E-F) Boxplots demonstrating the degree of significance (e-values) between N-terminal domains of antibody-bound flagellin peptides and N-terminal domains of reference sequences for stimulator-, silent-, and evader-type flagellin in patients with CD (left panel) and ME/CFS (right panel). (G-H) Boxplots demonstrating the degree of significance (e-values) between C-terminal domains of antibody-bound flagellin peptides and C-terminal domains of reference sequences for stimulator-, silent-, and evader-type flagellin in patients with CD (left panel) and ME/CFS (right panel). Supplemental Figure S2. Peptide sequences of antibody-bound flagellins overrepresented in CD and ME/CFS resemble full-length stimulator- and silent flagellins and highly (>2.5 fold) overrepresented full-length antibody-bound flagellins overrepresented in CD and ME/CFS resemble both stimulator- and silent flagellins. (A-B) Boxplots showing the degree of sequence similarity (identity%, left panel) and statistical significance (e-values, right panel) for peptide-length antibody-bound flagellins in CD towards reference sequences of stimulator-, silent-, and evader-type flagellin peptides. (C-D) Boxplots showing the degree of sequen [file 40168_2024_1858_MOESM2_ESM.docx]

## Supplementary Figure S1


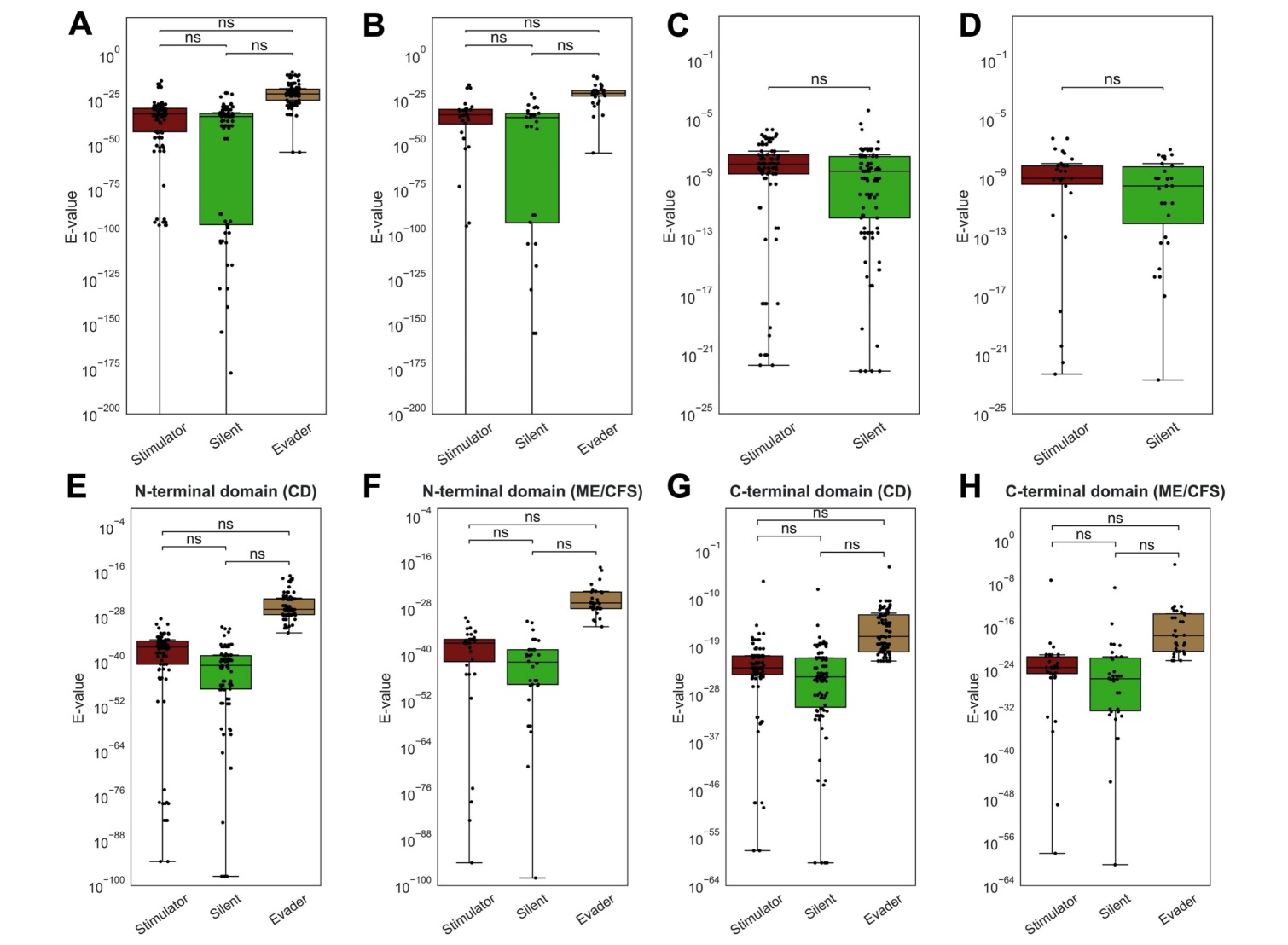


**Supplemental Figure S1. Sequences of antibody-bound flagellins overrepresented in CD and ME/CFS resemble both stimulator- and silent flagellins while C-terminal antibody-bound flagellin sequences exhibit higher resemblance to stimulator flagellins in CD.** (**A-B**) Boxplots showing the degree of statistical significance (e-values) for full-length antibody-bound flagellin peptides towards reference sequences of stimulator-, silent-, and evader-type flagellin peptides (panel A: CD; panel B: ME/CFS). (**C-D**) Boxplots demonstrating the degree of statistical significance (e-values) between full-length antibody-bound flagellin peptide sequences and reference motifs for the C-terminal D0 domain (containing the allosteric binding site) (panel C: CD; panel D: ME/CFS). (**E-F**) Boxplots demonstrating the degree of significance (e-values) between N-terminal domains of antibody-bound flagellin peptides and N-terminal domains of reference sequences for stimulator-, silent-, and evader-type flagellin in patients with CD (left panel) and ME/CFS (right panel). (**G-H**) Boxplots demonstrating the degree of significance (e-values) between C-terminal domains of antibody-bound flagellin peptides and C-terminal domains of reference sequences for stimulator-, silent-, and evader-type flagellin in patients with CD (left panel) and ME/CFS (right panel).

## Supplementary Figure S2


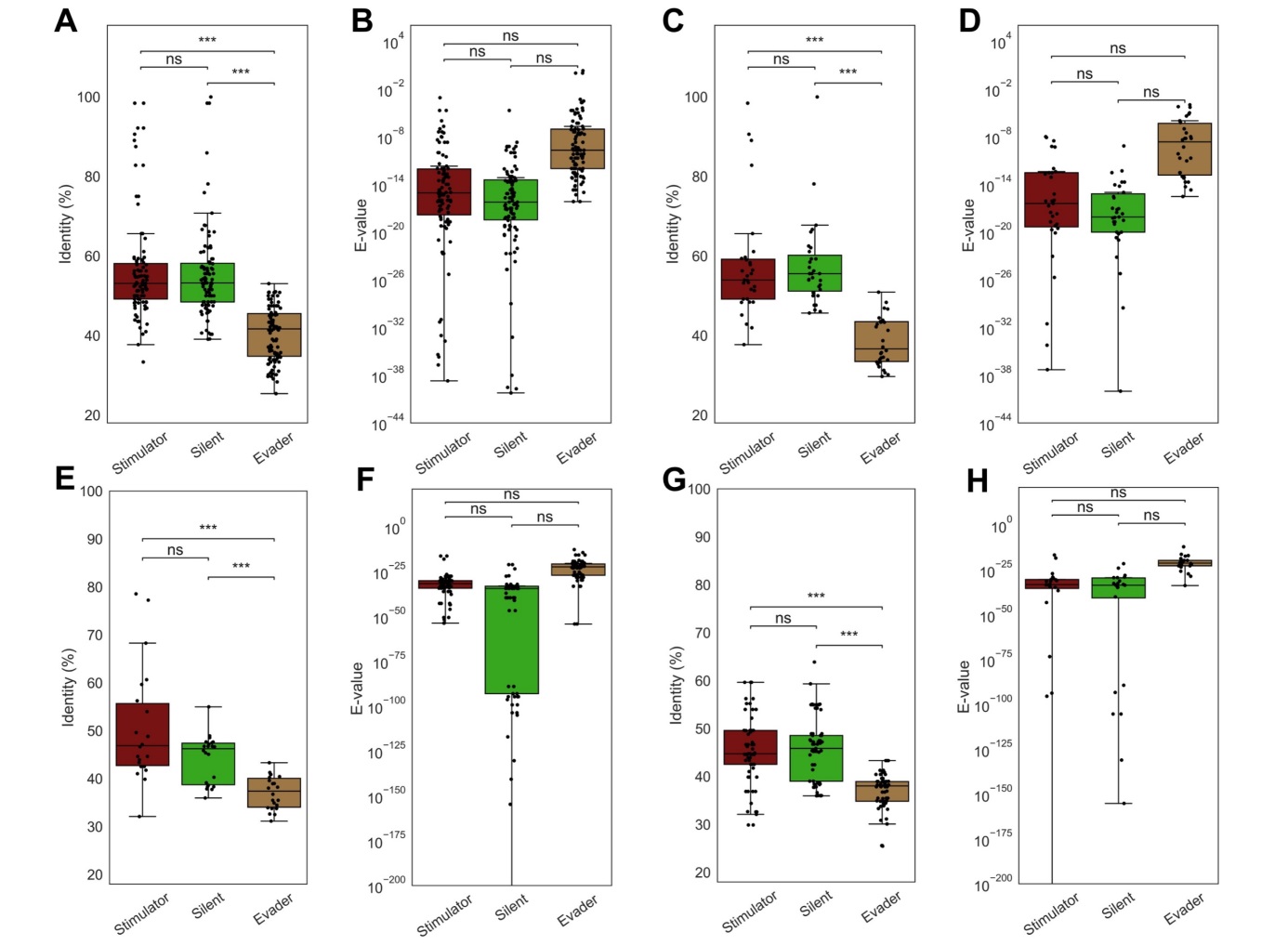


**Supplemental Figure S2. Peptide sequences of antibody-bound flagellins overrepresented in CD and ME/CFS resemble full-length stimulator- and silent flagellins and highly (>2.5 fold) overrepresented full-length antibody-bound flagellins overrepresented in CD and ME/CFS resemble both stimulator- and silent flagellins.** (**A-B**) Boxplots showing the degree of sequence similarity (identity %, left panel) and statistical significance (e-values, right panel) for peptide-length antibody-bound flagellins in CD towards reference sequences of stimulator-, silent-, and evader-type flagellin peptides. (**C-D**) Boxplots showing the degree of sequence similarity (identity %, left panel) and statistical significance (e-values, right panel) for peptide-length antibody-bound flagellins in ME/CFS towards full-length reference sequences of stimulator-, silent-, and evader-type flagellin peptides. (**E-F**) Boxplots showing the degree of sequence similarity (identity %, left panel) and statistical significance (e-values, right panel) for highly (>2.5 fold) overrepresented full-length antibody-bound flagellins in CD towards reference sequences of stimulator-, silent-, and evader-type flagellin peptides. (**G-H**) Boxplots showing the degree of sequence similarity (identity %, left panel) and statistical significance (e-values, right panel) for highly (>2.5 fold) overrepresented full-length antibody-bound flagellins in ME/CFS towards full-length reference sequences of stimulator-, silent-, and evader-type flagellin peptides.

## Supplementary Figure S3


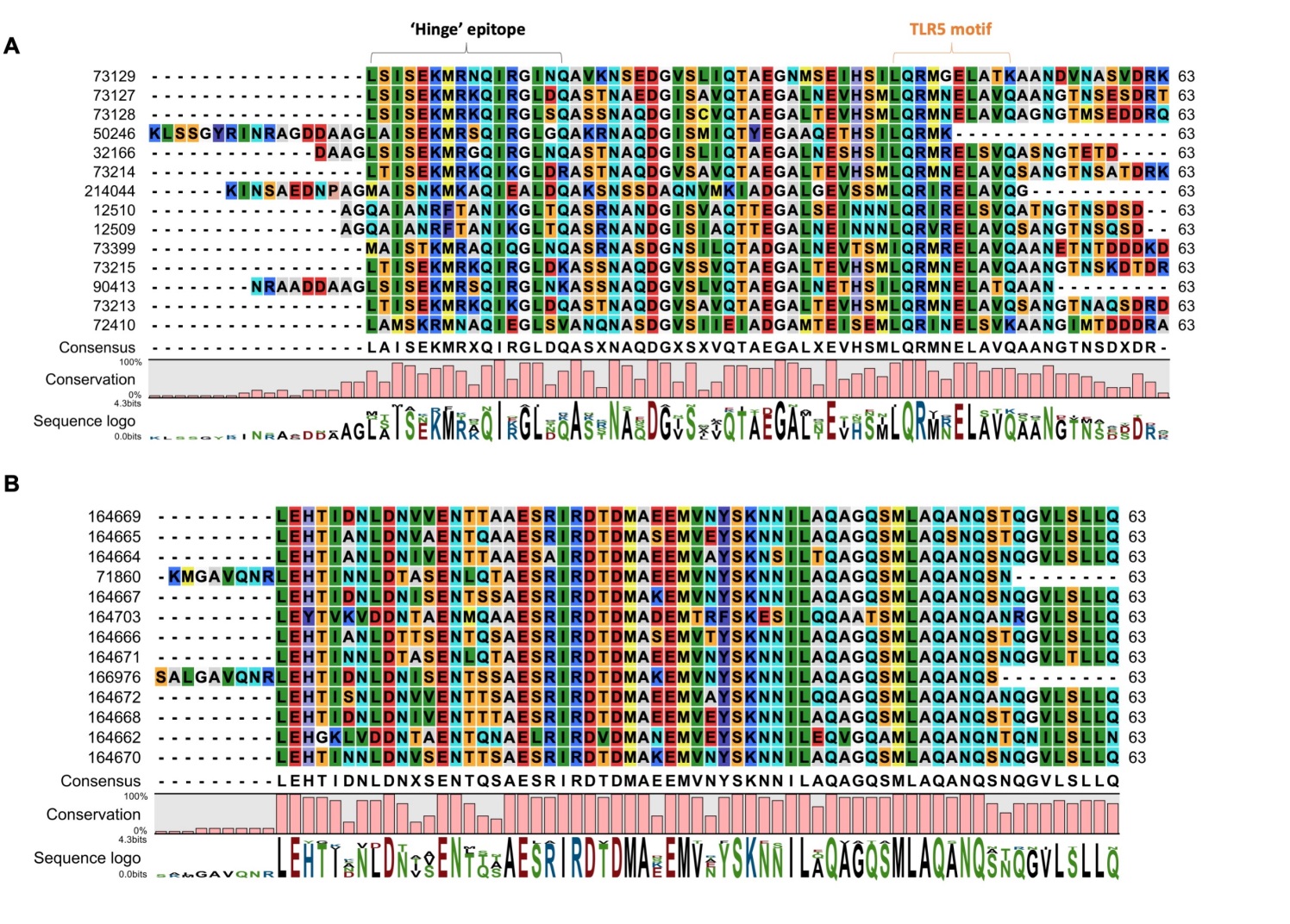


**Supplementary Figure S3. Full segment alignments of antibody-bound bacterial flagellins overrepresented in patients with CD and ME/CFS sharing distinct N- and C-terminal sequence motifs.** (**A**) Full alignment of subset of antibody-bound flagellin peptides overrepresented in both patients with CD and ME/CFS; the location of the hinge epitope (nD0-1) and TLR5 motif (nD1) are highlighted and show shared motifs putatively bound by antibody responses. (**B**) Full alignment of antibody-bound flagellin peptides that were >25 fold overrepresented in patients with CD which all share a common C-terminal motif putatively bound by antibody responses. Alignments were generated using MEGA software using the multiple sequence comparison by log-expectation (MUSCLE) algorithm in default settings (see Methods).

## Supplementary Figure S4


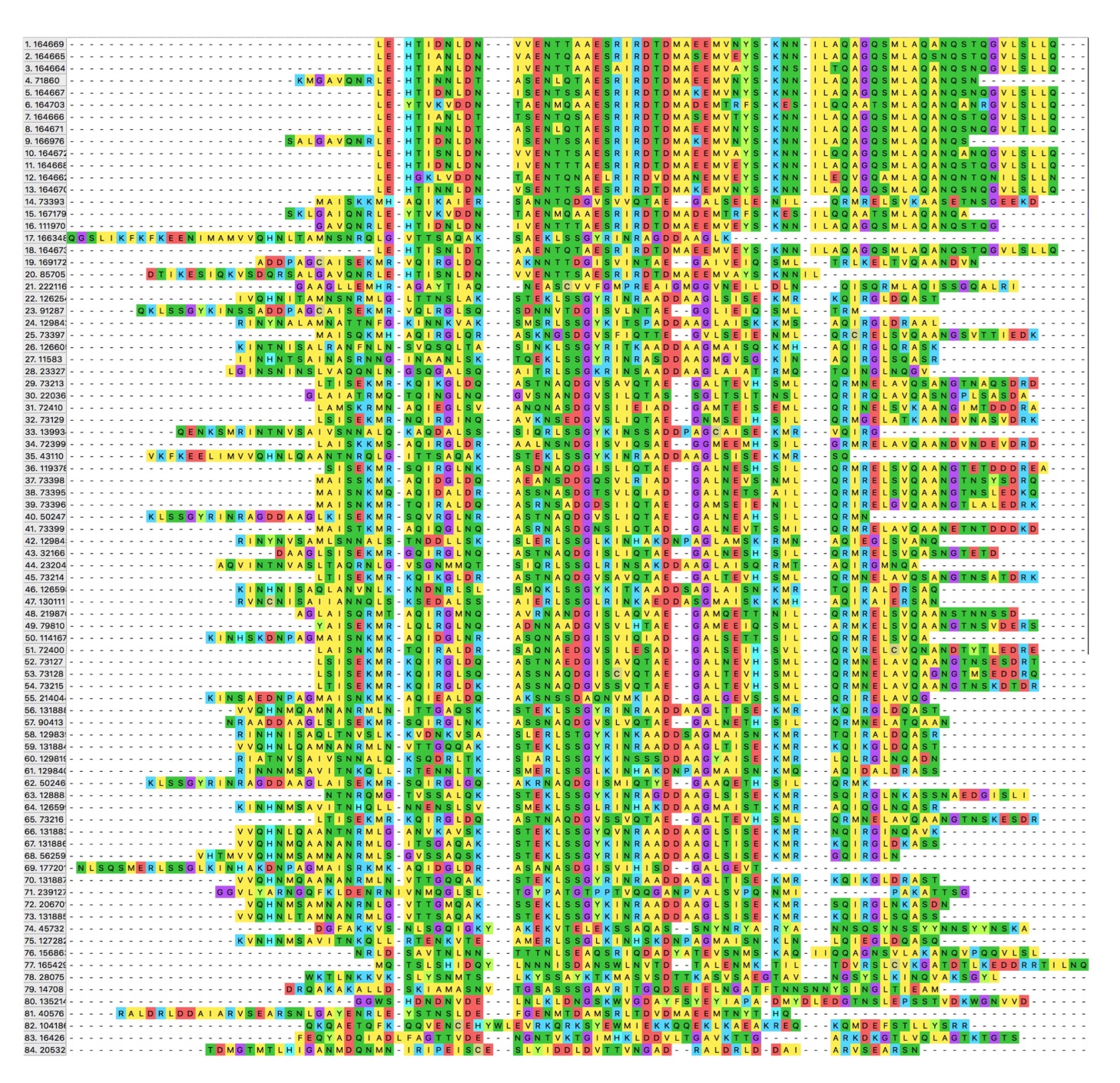


**Supplementary Figure S4. Full alignments of antibody-bound bacterial flagellins overrepresented in patients with CD showing both N- and C-terminal sequence motifs.** Shown is the full alignment of antibody-bound flagellin peptides overrepresented in patients with CD in which both the N-terminal motifs shared with patients with ME/CFS is observed (albeit in a different order, lower red rectangle) as well as the CD-specific C-terminal motif (upper red rectangle). Alignments were generated using MEGA software using the multiple sequence comparison by log-expectation (MUSCLE) algorithm in default settings (see Methods).

## Supplementary Figure S5


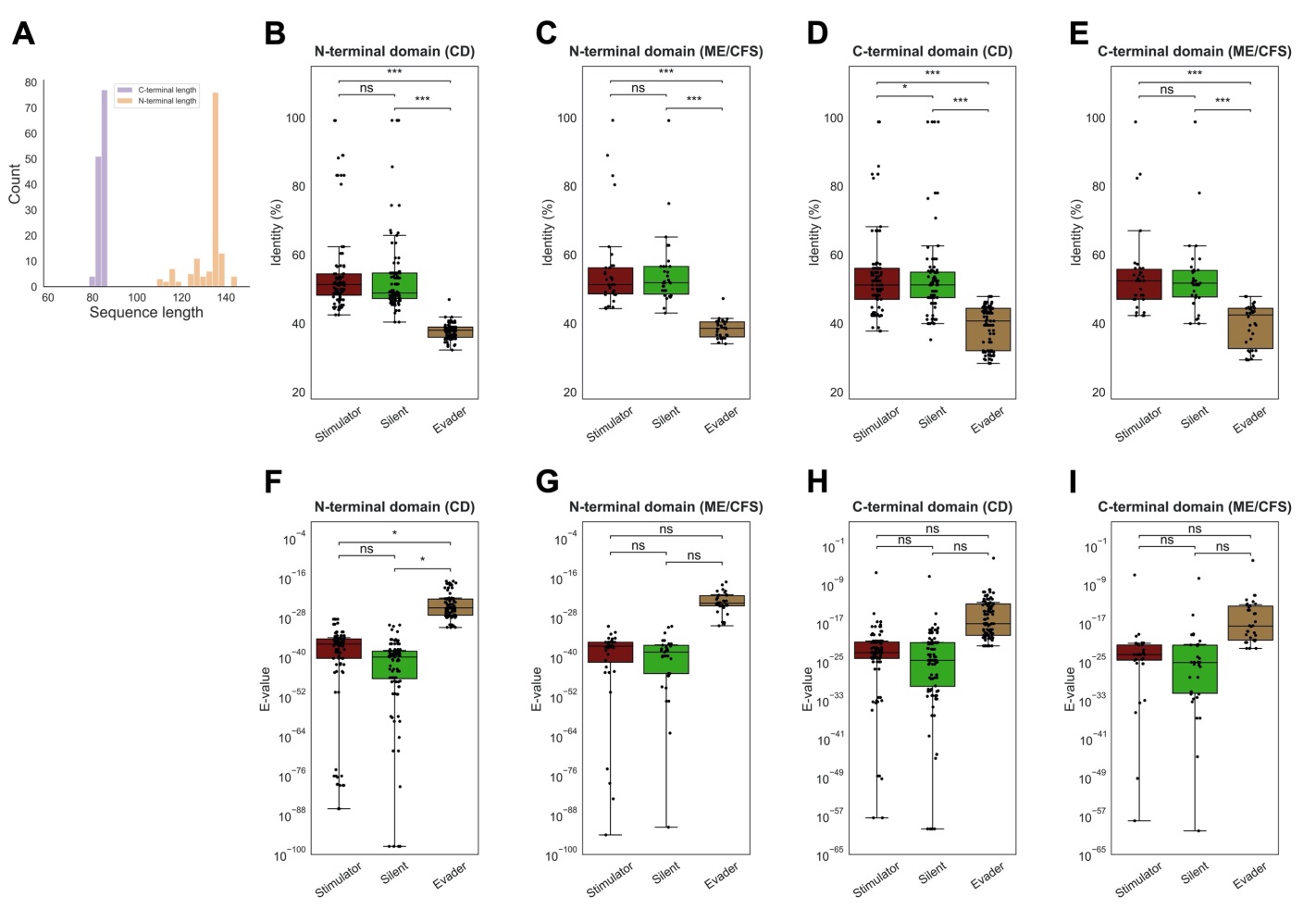


**Supplementary Figure S5. Sequences of antibody-bound flagellins overrepresented in CD and ME/CFS resemble both stimulator- and silent flagellins also using alternative domains for alignments.** (**A**) Histogram showing the distributions of sequence lengths of extracted and annotated N- and C-terminal domains of antibody-bound flagellin peptides when using the silent domain rather than the stimulator one as reference for the N-termini (see Methods). (**B-C**) Boxplots demonstrating the degree of sequence identity (%) between N-terminal domains of antibody-bound flagellin peptides and N-terminal domains of reference sequences for stimulator-, silent-, and evader-type flagellin in patients with CD and ME/CFS, yielding similar results as shown in Fig. 4, Fig. S1, and Fig. S2, demonstrating that the selection of the reference domain for alignments does not strongly impact outcomes. (**D-E**) Boxplots demonstrating the degree of sequence identity (%) between C-terminal domains of antibody-bound flagellin peptides and C-terminal domains of reference sequences for stimulator-, silent-, and evader-type flagellin in patients with CD and ME/CFS. (**F-G**) Boxplots demonstrating the degree of significance (e-values) between N-terminal domains of antibody-bound flagellin peptides and N-terminal domains of reference sequences for stimulator-, silent-, and evader-type flagellin in patients with CD (left panel) and ME/CFS (right panel). (**H-I**) Boxplots demonstrating the degree of significance (e-values) between C-terminal domains of antibody-bound flagellin peptides and C-terminal domains of reference sequences for stimulator-, silent-, and evader-type flagellin in patients with CD (left panel) and ME/CFS (right panel).

## Supplementary Figure S6


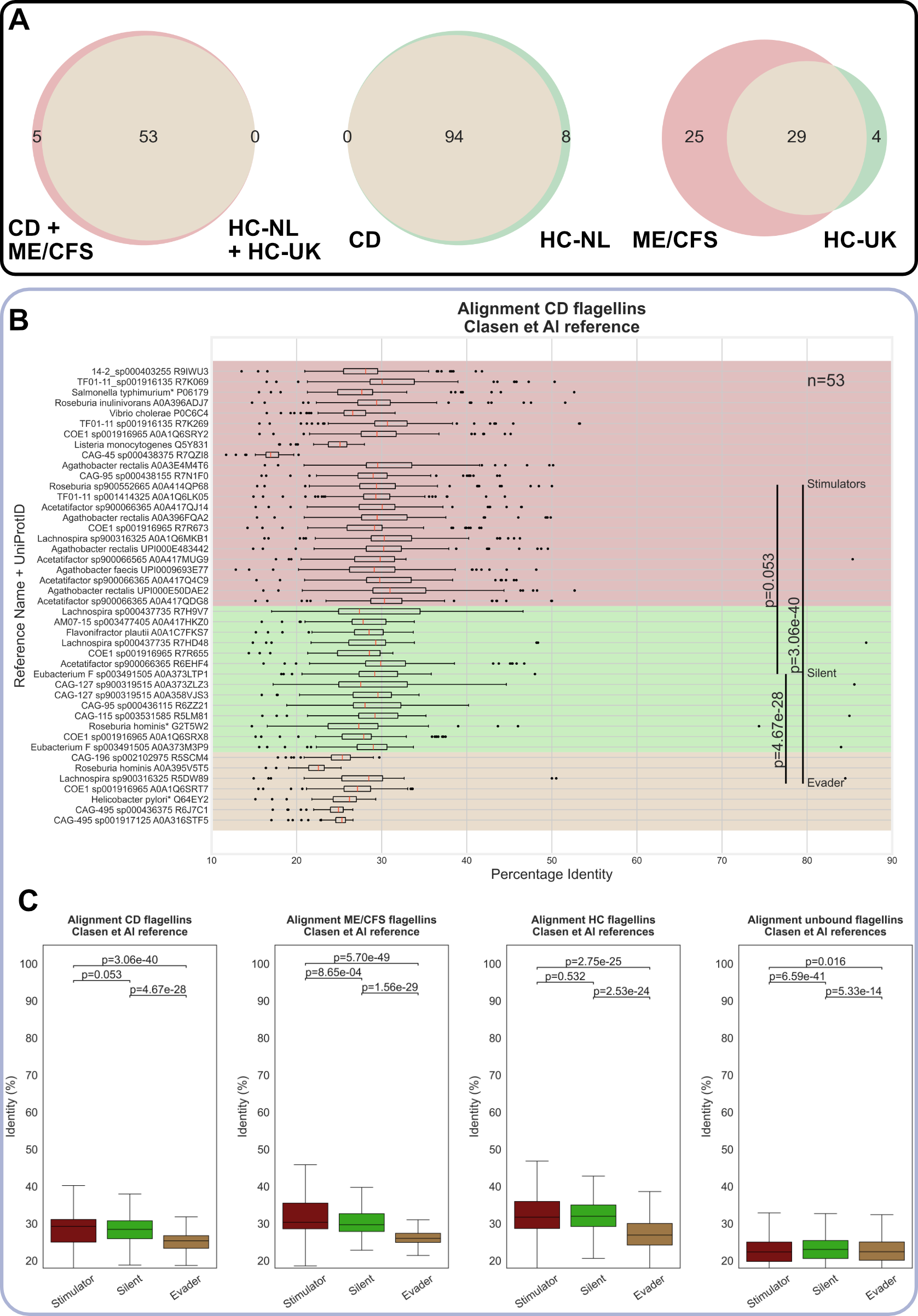


**Supplementary Figure S6. In contrast to unbound flagellins, the antibody bound flagellins in disease- and healthy control cohorts overlap in occurrence and resemble the stimulator and silent subgroups of 44 reference flagellins.** (**A**) Venn diagrams illustrating overlaps of the antibody bound flagellins in CD, ME/CFS, HC-NL and HC-UK. (**B**) Bar plot showing the degree of sequence identity (%) for full length antibody-bound flagellin peptides in CD towards each of the 44 reference sequences (**Table S4**). (**C**) Boxplots showing the summarized degree of sequence identity (%) for full length antibody-bound flagellin peptides in CD, ME/CFS, and the HCs as well as unbound flagellins towards the 44 reference sequences (Table S4). *P*-values for comparison of sequence identity (%) were derived from Kruskal Wally’s tests followed by Dunn’s post hoc tests with Bonferroni *P*-value adjustment.

## Supplementary Figure S7


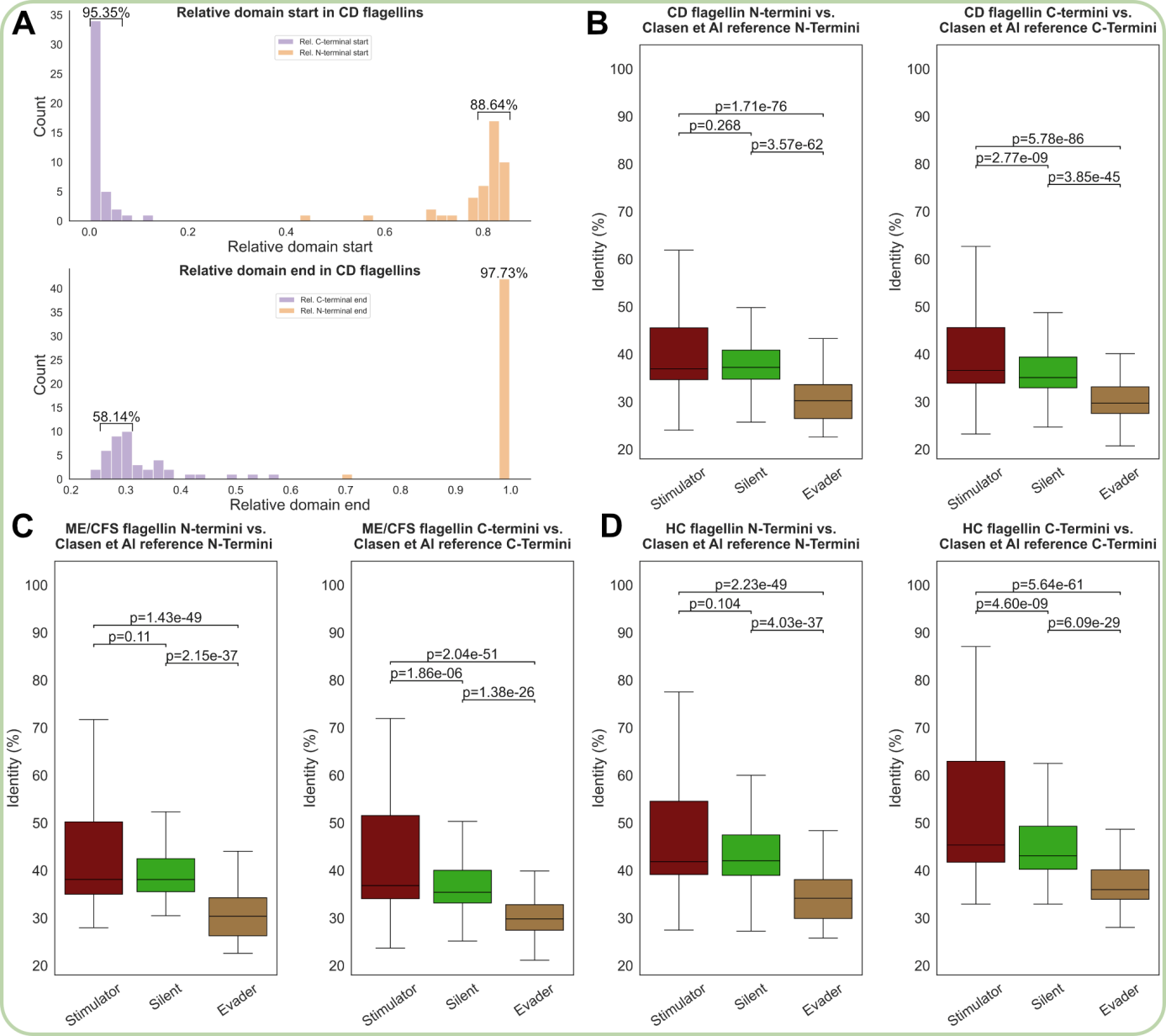


**Supplementary Figure S7. N- and C-terminal domain-sequences of antibody-bound flagellins overrepresented in CD and ME/CFS as well as domain sequences of flagellins antibody-bound in HCs resemble both stimulator- and silent flagellin group of 44 references while C-terminal antibody-bound flagellin sequences exhibit higher resemblance to stimulator flagellins in CD, ME/CFS and the HCs.**

(**A**) Histogram showing the distributions of the relative start and end positions of the N- and C-terminal domains of antibody-bound flagellins in CD. The brackets describe the ratio of all domain sequences in contained the marked bins. (**B**) Boxplots demonstrating the summarized degree of sequence identity (%) between N- and C-terminal domains of antibody-bound flagellin peptides and N- and C-terminal domains of 44 reference sequences (**Table S4**) in patients with CD. (**C**) Boxplots demonstrating the summarized degree of sequence identity (%) between N- and C-terminal domains of antibody-bound flagellin peptides and N- and C-terminal domains of 44 reference sequences (Table S4). Al in patients with ME/CFS. (**D**) Boxplots demonstrating the summarized degree of sequence identity (%) between N- and C-terminal domains of antibody-bound flagellin peptides and N- and C-terminal domains of 44 reference sequences (Table S4) in the healthy control cohorts. *P*-values for comparison of sequence identity (%) were derived from Kruskal Wallis tests followed by Dunn’s post hoc tests with Bonferroni *P*-value adjustment.
